# Macrophage-derived MCPIP1 mediates silica-induced pulmonary fibrosis via autophagy

**DOI:** 10.1186/s12989-016-0167-z

**Published:** 2016-10-25

**Authors:** Haijun Liu, Shencun Fang, Wei Wang, Yusi Cheng, Yingming Zhang, Hong Liao, Honghong Yao, Jie Chao

**Affiliations:** 1 Department of Physiology, School of Medicine, Southeast University, 87 Dingjiaqiao Rd, Nanjing, Jiangsu 210009 China; 2 Neurobiology Laboratory, New Drug Screening Centre, China Pharmaceutical University, Nanjing, Jiangsu 210009 China; 3 Nine Department of Respiratory Medicine, Nanjing Chest Hospital, Nanjing, Jiangsu 210029 China; 4 Department of Pharmacology, School of Medicine, Southeast University, 87 Dingjiaqiao Rd, Nanjing, Jiangsu 210009 China; 5 Key Laboratory of Developmental Genes and Human Disease, Southeast University, Nanjing, 210096 China; 6 Department of Respiration, Zhongda Hospital, School of Medicine, Southeast University, Nanjing, Jiangsu 210009 China

**Keywords:** Autophagy, MCPIP1, Silicosis, p53, Migration

## Abstract

**Background:**

Silicosis is characterized by accumulation of fibroblasts and excessive deposition of extracellular matrix. Monocyte chemotactic protein-1-induced protein 1 (MCPIP1) plays a critical role in fibrosis induced by SiO_2_. However, the details of the downstream events of MCPIP1 activity in pulmonary fibrosis remain unclear. To elucidate the role of MCPIP1-induced autophagy in SiO_2_-induced fibrosis, both the upstream molecular mechanisms and the functional effects of SiO_2_ on cell apoptosis, proliferation and migration were investigated.

**Results:**

Experiments using primary cultures of alveolar macrophages from healthy donors and silicosis patients as well as differentiated U937 macrophages demonstrated the following results: 1) SiO_2_ induced macrophage autophagy in association with enhanced expression of MCPIP1; 2) autophagy promoted apoptosis and activation of macrophages exposed to SiO_2_, and these events induced the development of silicosis; 3) MCPIP1 facilitated macrophage apoptosis and activation via p53 signaling-mediated autophagy; and 4) SiO_2_-activated macrophages promoted the proliferation and migration of fibroblasts via the MCPIP1/p53-mediated autophagy pathway.

**Conclusions:**

Our results elucidated a link between SiO_2_-induced fibrosis and MCPIP1/p53 signaling-mediated autophagy. These findings provide novel insight into the potential targeting of MCPIP1 or autophagy in the development of potential therapeutic strategies for silicosis.

**Electronic supplementary material:**

The online version of this article (doi:10.1186/s12989-016-0167-z) contains supplementary material, which is available to authorized users.

## Background

Occupational exposure to silica dust occurs in many industries and leads to reduced lung function characterized by excessive fibroblast proliferation and collagen deposition, ultimately causing respiratory failure [1], which is a serious problem in developing and even developed countries. However, the exact etiology of silicosis is not well understood.

Macrophages, the first line of defense against silica dust, play a crucial role in the development of silicosis [2, 3]. Upon interacting with silica, macrophages engulf the dust particles, which are then removed from the lungs through the mucociliary clearance system [4]. However, when macrophages fail to dissolve the crystalline structure of the silica, continuously activated macrophages initiate a cascade of responses that contribute to inflammatory reactions and the development of fibrosis, especially after the activated macrophages migrate to the interstitial space [5, 6]. Macrophages show typical characteristics of heterogeneity and plasticity [7]. When macrophages respond to external stimuli, they become functionally polarized into different phenotypes, specifically the classically activated (M1) and alternatively activated (M2) phenotypes [8]. Many studies have indicated that M2 macrophages, which are characterized by increased expression of the effector proteins YM1, FIZZ1 and Arginase 1 [9], are involved in tissue repair and regeneration during the anti-inflammatory phase [10, 11]. This expression profile creates a microenvironment that promotes the development of fibrosis, driving the proliferation, migration, and transdifferentiation of mesenchymal cells. In particular, interstitial macrophages activated by invasive stimuli produce growth-promoting cytokines that provoke proliferative signaling by fibroblasts and ultimately induce alterations in collagen metabolism and deposition in the lungs [12, 13].

Autophagy, an evolutionarily conserved process, plays a key role in the maintenance of cell homeostasis by degrading misfolded or dysfunctional proteins [14] and even organelles such as peroxisomes and mitochondria [15, 16]. Autophagy contributes to the removal of toxic intracellular substances and promotes cell survival by generating recycled products. Alternatively, autophagy may also participate in irreversible cell injury and cell death under extreme conditions [17, 18]. Autophagy appears to be involved in the development of several lung diseases, such as chronic obstructive pulmonary disease (COPD) [19], cystic fibrosis (CF) [20], pulmonary arterial hypertension (PAH) [21], lung cancer [22] and idiopathic pulmonary fibrosis (IPF) [23]. However, very few studies of autophagy have investigated its relation to silicosis, let alone the role of autophagy by macrophages in silicosis. A wealth of evidence suggests that nanoparticles may be sequestered by autophagosomes. In addition, autophagosomes could selectively engulf invading nanomaterials. Treatment of murine macrophages or human lung adenocarcinoma cells with silica nanoparticles has been suggested to promote the formation of autophagosomes possessing a double-membrane structure [24, 25]. Recent studies [26] have indicated that the dysregulation of autophagy in histiocytes of granulomas may contribute to granuloma development and progression in silicosis. It is possible that aberrant autophagy plays an important role in the pathogenesis of silicosis, but the exact molecular mechanisms by which autophagy is activated via silica exposure remain unknown.

Monocyte chemotactic protein-1 (MCP-1), also referred to as C-C chemokine ligand 2, CCL2), is expressed by various cell types, such as macrophages, fibroblasts, endothelial cells and epithelial cells [27–30]. MCP-1 exhibits increased expression in silicosis [31]. C-C chemokine receptor 2 (CCR2) is a receptor for a few CCL2 family members, including CCL2. The binding of CCL2 to CCR2 activates the transduction of signals to downstream targets [32]. Our recent study indicated that the MCP-1/CCR2 signaling pathway plays a major role in SiO_2_-induced pulmonary fibroblast migration [33]. MCP-1-induced protein 1 (MCPIP1), a novel zinc finger protein, is a newly discovered protein induced by MCP-1 in human peripheral blood monocytes [34]. Recent reports suggest that MCPIP1 mediates numerous cellular processes, including the regulation of gene transcription [34], mRNA degradation [35], cell apoptosis [36, 37], autophagy [38] and differentiation [39], through its activities as a transcription factor, RNase, or deubiquitinase. Although MCPIP1 is recognized as the pivotal downstream molecule of MCP-1, it is unknown whether MCPIP1 mediates SiO_2_-induced silicosis, and the molecular mechanisms involved in MCPIP1-mediated silicosis have not been identified.

In this study, we show that increased expression of MCPIP1 causes autophagy, leading to the activation and death of macrophages through MCPIP1/p53-mediated autophagic signaling. These findings identify a novel function of MCPIP1-mediated autophagy in SiO_2_-induced fibrosis and suggest that MCPIP1 may be involved in multiple steps of the fibrosis process.

## Methods

### Reagents

Silicon dioxide, 80 % of which had a particle diameter of less than 5 μm, was purchased from Sigma® (S5631) (Additional file 1: Table S1), selected via sedimentation according to Stokes’ law, acid-hydrolyzed, and baked overnight (200 °C, 16 h). The silica samples for the cell experiments were suspended in normal saline (NS) at a concentration of 5 mg/ml, and the volume applied was 20 μl/well in a 24-well plate, corresponding to a silica dosage of 50 μg/cm^2^. Fetal bovine serum (FBS), normal goat serum (NGS) and Dulbecco's modified Eagle's medium (DMEM; #1200–046) were purchased from Life Technologies™. Amphotericin B (BP2645) and GlutaMAX™ supplement (35050–061) were obtained from Gibco®, and Pen/Strep (15140–122) was obtained from Fisher Scientific. Antibodies against MCPIP1 (SC136750, goat), p53 (SC6243, rabbit) and β-actin (SC8432, mouse) were obtained from Santa Cruz Biotechnology®, Inc. The antibody against α-SMA (SAB5500002) was purchased from Sigma, Inc. The short interfering RNA (siRNA) transfection reagent (SC29528) and MCPIP1 siRNA (SC78944) were purchased from Santa Cruze Biotechnology®, Inc.

### Cell culture

The human monocytic cell line U937 (ATCC) was cultured at 8 × 10^5^ cells/well in RPMI 1640 medium containing 10 % FBS, penicillin (50 U/ml) and streptomycin (100 μg/ml) at 37 °C in a 5 % CO_2_ atmosphere. Then, 50 nM phorbol myristate acetate (PMA) was used to differentiate U937 cells for 24 hours prior to the experiments.

Human pulmonary fibroblasts (ScienCell) were cultured in DMEM supplemented with 10 % FBS, 100 U/ml penicillin, 100 μg/ml streptomycin and 2 mM L-GlutaMAX (obtained from Gibco®) at 37 °C in a humidified 5 % CO_2_ atmosphere.

### Western blotting

Treated cells were washed three times with cold PBS and then lysed using a mammalian cell lysis kit (MCL1-1KT, Sigma-Aldrich®). Electrophoretic analysis of equal amounts of the proteins was performed via SDS-PAGE (12 %) under reducing conditions. The proteins that were separated via gel electrophoresis were transferred to PVDF membranes and then blocked with 5 % non-fat dry milk in TBST at room temperature for 1 h. The membranes were incubated overnight at 4 °C with the indicated antibodies and then incubated with an alkaline phosphatase-conjugated goat anti-mouse or anti-rabbit IgG secondary antibody (1:5000 dilution) in TBS-T for 1 h at room temperature. A chemiluminescence detection system was used to detect the immunoreactive protein bands. The intensity of the protein bands was normalized to the corresponding intensity of the internal control via densitometry using ImageJ software (NIH). Each western blot was repeated at least three times.

### Immunoprecipitation

Macrophages were collected in RIPA lysis buffer (Beyotime, Nantong, China), and the protein concentrations were determined using a BCA Protein Assay Kit (Pierce, Rockford, IL). Equal amounts of the proteins were incubated with an anti-p53 antibody overnight at 4 °C, followed by incubation with 20 μl of protein A Sepharose for 90 min at 4 °C. The mixture was centrifuged (12,000 rpm, 1 min, 4 °C), and the cell pellets were rinsed twice with RIPA lysis buffer. The cell pellets were boiled in 5× western blot loading buffer and RIPA lysis buffer for 5 min. After centrifugation (12,000 rpm, 1 min), the supernatants were subjected to western blot for the detection of MCPIP1.

### Cell migration assays

Cell migration assays were used to determine the motility of the fibroblasts as previously described [33]. Briefly, HPF-a cells were seeded in 24-well culture plates at 1 × 10^5^ cells/well and cultured in growth medium until reaching approximately 70–80 % confluence. Then, the cell monolayer was gently scratched with a sterile 200-μl pipette tip to generate a wide gap. Using fresh growth medium, each well was washed twice to remove the cell debris. Conditioned media was applied to continue cell growth for 24 h. Images of the scratch were captured at 0 and 24 h, and the width of the cell gap was quantified using ImageJ software.

### 3-(4,5-dimethylthiazol-2-yl)-2,5-diphenyltetrazolium bromide (MTT) assays

Cell viability was measured using MTT assays. Briefly, the cells were seeded in 96-well plates at a density of 5 × 10^4^ cells/well (for macrophages) or 2 × 10^4^ cells/well (for HPF-a cells) and cultured in an incubator containing 5 % CO_2_ at 37 °C for 24 h. The cells were treated with 50 μg/cm^2^ SiO_2_ for 0, 6, 12, or 24 h (for macrophages) or with the indicated conditioned media (for fibroblasts). Following culture for 24 h, freshly prepared MTT solution was applied to the treated cells for 2–4 h at 37 °C. Then, the cell supernatant was removed using a vacuum pump, and the cells were treated with 150 μl of dimethyl sulfoxide to dissolve the formed formazan crystals. To fully dissolve the formazan crystals, the 96-well plate was placed on a shaker for 10 min. Afterwards, the absorbance of each well was measured at a wavelength of 490 nm using a BioTek microplate reader (SYNERGY H1; BioTek, Highland Park, VT, USA).

### Human bronchoalveolar lavage fluid (BALF)

Human BALF was obtained from Nanjing Chest Hospital. The use of primary alveolar macrophages derived from human BALF was performed in accordance with the approved guidelines of the Research and Development Committee of Nanjing Chest Hospital. After filtering the BALF through a multilayer gauze, the BALF was centrifuged in a 50-ml centrifuge tube at 4 °C for 10 min at 1800 rpm. The cells were resuspended in serum-free medium after the supernatant was discarded. Then, the cells were counted with a hemocytometer and seeded in a 24-well plate at 5 × 10^5^ cells/well. After incubating the cells for 2 h at 5 % CO_2_ and 37 °C, the serum-free medium was removed from the plate, and each well was washed twice with cold PBS to remove non-adherent cells and cell debris. The cells, 95 % of which were macrophages, cultured in complete medium were used for further experiments.

### Small interference RNA (siRNA)-mediated knockdown

Macrophages were transfected with siRNA to knock down the protein levels of MCPIP1or p53 to determine their downstream signaling activity. Briefly, U937 cells were seeded at 8 × 10^5^ cells/well in a 24-well plate, and 50 nM PMA was applied as indicated. To knock down protein expression, chemically synthesized siRNA targeting MCPIP1or p53 was transfected into macrophages using Lipofectamine 2000 according to the manufacturer’s instructions (Santa Cruz Biotechology®); a non-specific siRNA was used as a negative control. For this purpose, the cells were incubated in serum-free DMEM containing siRNA combined with Lipofectamine 2000 for 18 h. Then, complete medium substituted for the serum-free DMEM, and the cells were cultured for an additional 24 h prior to the subsequent experiments.

### Lentiviral transfection

P3-4 primary human pulmonary fibroblasts (HPF-a cells) were transfected with LV-RFP lentivirus (HANBIO Inc., Shanghai, China) as previously described [40]. Briefly, HPF-a cells were seeded at 1 × 10^4^ cells/well in a 24-well plate in DMEM containing 10 % FBS and were cultured for 48 h. The medium was replaced with 1 ml of fresh medium containing 8 μg/ml polybrene. Subsequently, the cells were incubated for 24 h, after which 100 μl of lentivirus solution (10^7^ IU/ml) was added to each well. After incubation, the lentivirus-containing medium was replaced with fresh DMEM containing 10 % FBS, and the cells were further incubated until reaching >50 % confluence. Using blasticidin, transduced cells were selected as follows. Briefly, the medium was replaced with DMEM containing 10 μg/ml puromycin and 10 % FBS, and the cells were cultured at 37 °C in 5 % CO_2_ for 24 h. Then, the cells were washed twice with fresh medium. Purified transduced HPF-a cell cultures were expanded and/or stored in liquid nitrogen as described previously [41].

### Hoechst 33342 staining

U937 cells were seeded on coverslips in 24-well plates and treated with PMA for 24 h. After differentiating into macrophages, the cells were stained with Hoechst 33342 (10 μg/ml) for 5 min at room temperature following different treatments. The populations of apoptotic cells were visualized under a fluorescence microscope. Five fields per well were randomly selected for apoptotic cell counting.

### ELISA

Supernatant from macrophages was collected, and the levels of MCP-1, TNF-α and TGF-β in the supernatants were determined using ELISA kits (SenBeiJia Biological Technology Co., Ltd., Nanjing, China) according to the manufacturer’s instructions.

### Immunofluorescence staining

Treated cells that were cultured on coverslips were washed twice with PBS and fixed with 4 % paraformaldehyde in PBS overnight at 4 °C. After two additional washes, the coverslips were incubated with 10 % NGS in 0.3 % Triton X-100 at room temperature for 2 h. Primary antibodies were incubated at 4 °C overnight. Then, the cells were incubated with the appropriate fluorescent secondary antibodies (1:250), and the nuclei were stained with 4’,6-diamidino-2-phenylindole (DAPI). Cell images were captured using a fluorescence microscope (Olympus IX70, Olympus America, Inc., Center Valley, PA, USA).

### Statistics

The data are presented as the means ± SEM. Unpaired numerical data were compared using an unpaired *t*-test (two groups) or analysis of variance (ANOVA; more than two groups). A p value of <0.05 was regarded as significant.

## Results

### Autophagy was induced in macrophages after exposure to silica

**Fig. 1:**
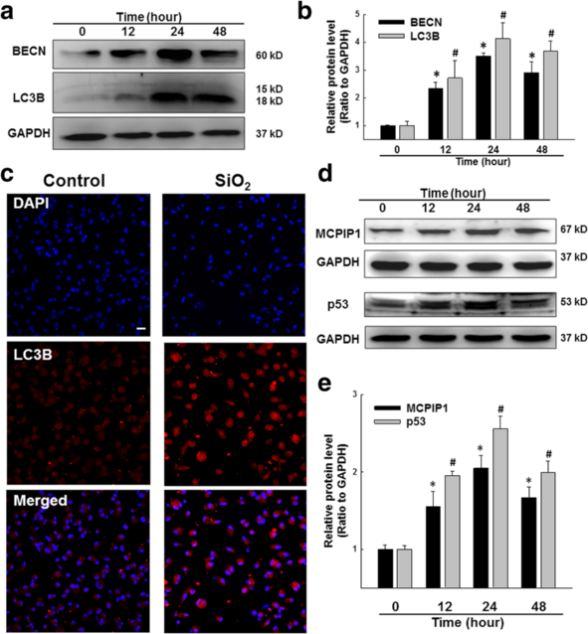
SiO_2_ induced autophagy via MCPIP1. **a** Representative western blot showing the effects of SiO_2_ (50 μg/cm^2^) on the expression of the autophagy markers BECN and LC3B in U937 cells. **b** Densitometric analyses of five separate experiments suggested that SiO_2_ induced increases in BECN and LC3B expression. *p < 0.05 vs BECN expression at 0 h; #*p* < 0.05 vs LC3B expression at 0 h. **c** Representative immunocytochemical images showing that SiO_2_ (50 μg/cm^2^) increased the expression of LC3B in U937 cells at 24 h following SiO_2_ treatment. Scale bar = 20 μm. **d** Representative western blot showing the effects of SiO_2_ on the expression of MCPIP1 and p53 in U937 cells. **e** Densitometric analyses of five separate experiments suggested that SiO_2_ induced increases in MCPIP1 and p53 expression. *p < 0.05 vs MCPIP1 expression at 0 h; #*p* < 0.05 vs p53 expression at 0 h

Recent reports have indicated that autophagy is induced in animal models of silicosis [26]. However, whether autophagy in macrophages is involved in the development of silicosis remains unknown. In this study, macrophages that were differentiated from U937 cells were used to study the effects of silica exposure on pulmonary cells. Because SiO_2_ cannot be dissolved and ultimately sinks to the bottom of the culture plate, micrograms per square centimeter (μg/cm^2^) was used as the unit of measure for the SiO_2_ dosage applied to cells. Previous studies [42, 43] have demonstrated that exposure of macrophages to SiO_2_ at 12.5-50 μg/cm^2^ induced cellular apoptosis based on the appearance of a series of indicators, such as apoptotic DNA fragmentation, mitochondrial depolarization, cytoplasmic shrinkage, and caspase 3 activation. We first examined the effect of SiO_2_ dosage (0, 12.5, 25, 50, 100, or 200 μg/cm^2^) on U937 macrophage autophagy. As shown in Additional file 2: Figure S1 A-B, SiO_2_ induced the expression of LC3B and BECN, markers of autophagy, in a dose-dependent manner. The maximal effect of SiO_2_ on autophagy marker expression was observed at 50 μg/cm^2^, a dosage that has been used in previous studies of silicosis [42, 43]. We chose 50 μg/cm^2^ for all subsequent experiments. Moreover, SiO_2_ induced significant increases in LC3B and BECN expression (Fig. 1a-b). Notably, the expression of autophagy markers peaked at 24 h. Similar results were obtained via immunofluorescence analysis, which showed significantly increased expression of LC3B following silica exposure (Fig. 1c).

### SiO_2_ induced MCPIP1 and p53 expression in U937 cells

Considering that previous studies have shown a relationship between MCPIP1 and autophagy under different pathological conditions [38, 44], we next measured the expression of MCPIP1 after SiO_2_ exposure in U937 cells. As shown in Fig. 1d-e, SiO_2_ induced the expression of MCPIP1, which peaked at 24 h. Previous data from our lab have shown an interaction between MCPIP1 and p53 in a different setting [39, 45]. Therefore, we also measured p53 expression after exposure to SiO_2_. As shown in Fig. 1d-e, SiO_2_ induced p53 expression, and this result was confirmed via immunocytochemistry (Additional file 3: Figure S2). Moreover, immunocytochemistry suggested co-localization of MCPIP1 with p53 in U937 cells after SiO_2_ exposure.

### MCPIP1 functioned via p53 signaling to mediate autophagic processes in macrophages exposed to silica

**Fig. 2:**
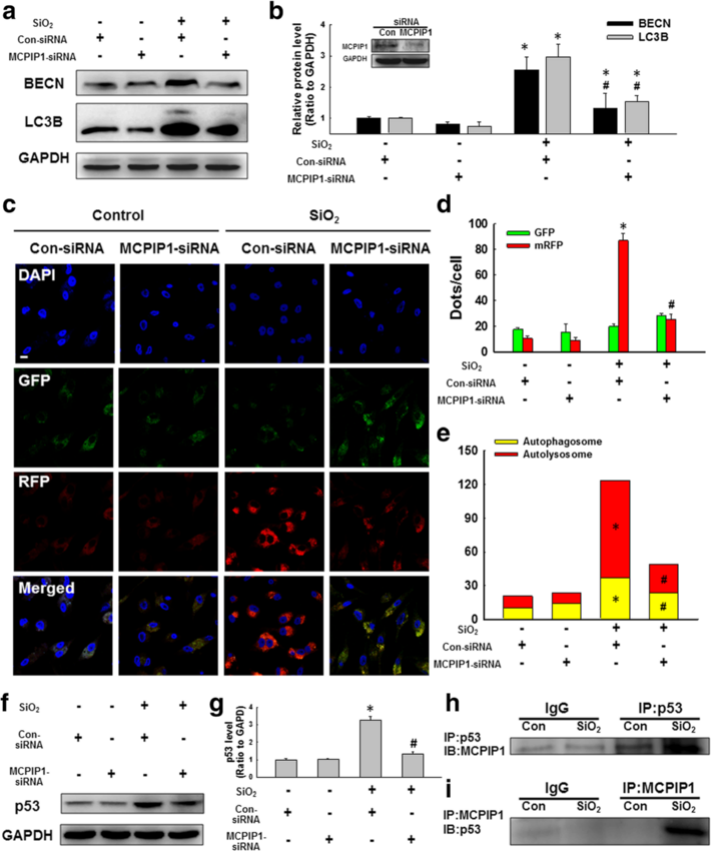
SiO_2_ induced cell apoptosis via autophagy. **a** Representative western blot showing the effects of RNAi targeting MCPIP1 on BECN and LC3B expression induced by SiO_2_ (50 μg/cm^2^) treatment for 24 h. **b** Densitometric analyses of five separate experiments suggested that SiO_2_-induced BECN and LC3B expression was attenuated by RNAi targeting MCPIP1. *p < 0.05 vs the corresponding control group; #*p* < 0.05 vs the corresponding SiO_2_ group. **c** Representative images showing the effect of RNAi targeting MCPIP1 on the SiO_2_-induced formation of RFP- and GFP-MAP1LC3 puncta at 24 h following SiO_2_ treatment. Scale bar = 10 μm. **d** Quantification of the RFP- and GFP- MAP1LC3 puncta demonstrating that SiO_2_-induced autophagy was attenuated by RNAi targeting MCPIP1. *p < 0.05 vs the control group; # *p* < 0.05 vs the SiO_2_ group. **e** Quantification of RFP-MAP1LC3 puncta and RFP- and GFP-colocalized puncta demonstrating autophagic flux induced by SiO_2_ was attenuated by RNAi targeting MCPIP1. *p < 0.05 vs the control group; #*p* < 0.05 vs the SiO_2_ group. **f** Representative western blot showing the effects of RNAi targeting MCPIP1 on SiO_2_-induced p53 expression. **g** Densitometric analyses of five separate experiments suggested that SiO_2_-induced p53 expression was attenuated by RNAi targeting MCPIP1. *p < 0.05 vs the control group; # *p* < 0.05 vs the SiO_2_ group. Whole cell lysates immunoprecipitated for p53 (**h**) or MCPIP1 (**i**) were then immunoblotted for MCPIP1 or p53, respectively, to determine the interaction between MCPIP1 and p53 after SiO_2_ exposure; IgG was used as an immunoprecipitation control. IP = immunoprecipitation; IB = immunoblot

To further understand the relationship between MCPIP1 and autophagy under conditions of SiO_2_-induced macrophage activation, in our study, MCPIP1-specific siRNA was applied. As shown in Fig. 2a-b, silencing MCPIP1 reduced the expression of autophagy markers, as indicated by a decrease in the LC3B and BECN levels compared to scramble control siRNA treatment. To validate our finding, a dual-fluorescence LC3-harboring adenovirus that is used to detect cell autophagy (mRFP-GFP-LC3) was applied to monitor the occurrence of autophagy in real time via fluorescence microscopy. mRFP was used to label and track LC3. Attenuated GFP fluorescence indicated fusion between a lysosome and an autophagosome to form an autolysosome because GFP fluorescence is sensitive to acidity, which quenches GFP fluorescence. As a consequence, only red fluorescence was detected under the microscope when autophagic flux was induced. As shown in Fig. 2c-e, SiO_2_ significantly induced autophagic flux, and this effect of SiO_2_ was attenuated by silencing MCPIP1.

To determine whether MCPIP1 functions through p53 signaling in silicosis, MCPIP1-specific siRNA was applied, and this treatment significantly decreased SiO_2_-induced p53 expression (Fig. 2f-g). Furthermore, SiO_2_ significantly enhanced the association of p53 with MCPIP1 based on immunoprecipitation assays (Fig. 2h-i). In addition, silencing p53 with a specific siRNA reduced the expression autophagy markers (data not shown), and this result was consistent with previous findings [46].

### Autophagy was responsible for the activation of macrophages in response to silica

**Fig. 3:**
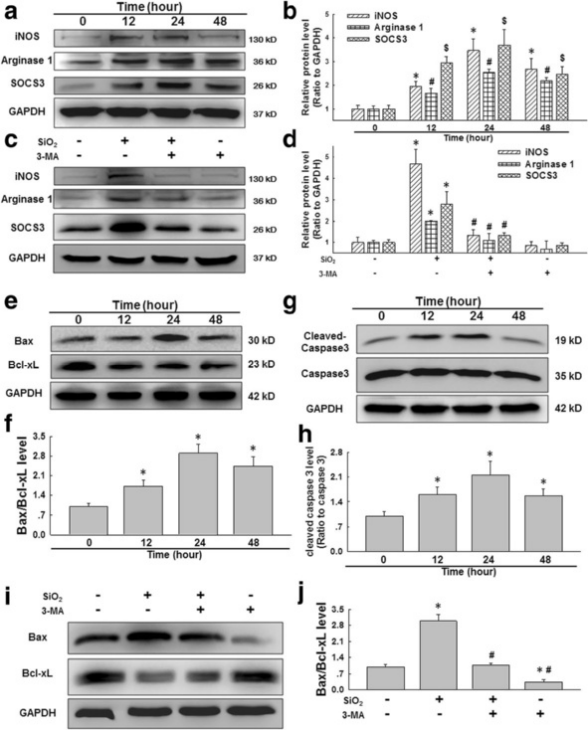
SiO_2_ induced macrophage activation via autophagy. **a** Representative western blot showing the effects of SiO_2_ (50 μg/cm^2^) on the expression of the M1 marker iNOS, the M2a marker Arginase 1 and the M2c marker SOCS3 in U937 cells. **b** Densitometric analyses of five separate experiments suggested that SiO_2_ induced iNOS, Arginase 1 and SOCS3 expression in a time-dependent manner. *p < 0.05 vs iNOS expression at 0 h; #*p* < 0.05 vs Arginase 1 expression at 0 h; $ *p* < 0.05 vs SOCS3 expression at 0 h. **c** Representative western blot showing the effects of 3-MA on the SiO_2_-induced expression of iNOS, Arginase 1 and SOCS3 in U937 cells at 24 h following 50 μg/cm^2^ SiO_2_ treatment. **d** Densitometric analyses of five separate experiments suggested that 3-MA attenuated the SiO_2_-induced increases in iNOS, Arginase 1 and SOCS3 expression. *p < 0.05 vs the corresponding control group; #*p* < 0.05 vs the corresponding SiO_2_ group. **e** Representative western blot showing the effects of SiO_2_ on the expression of Bax and Bcl-xL in U937 cells. **f** Densitometric analyses of five separate experiments suggested that SiO_2_ induced Bax/Bcl-xL expression in a time-dependent manner. *p < 0.05 vs the Bax/Bcl-xL levels at 0 h. **g** Representative western blot showing the effects of SiO_2_ on the expression of cleaved caspase-3 and total caspase-3 in U937 cells. **h** Densitometric analyses of five separate experiments suggested that SiO_2_ induced cleaved caspase-3/total caspase-3 expression in a time-dependent manner. *p < 0.05 vs the cleaved caspase-3/total caspase-3 levels at 0 h. **i** Representative western blot showing the effects of the specific autophagy inhibitor 3-MA on the SiO_2_-induced expression of Bax and Bcl-xL in U937 cells. **j** Densitometric analyses of five separate experiments suggested that 3-MA attenuated the SiO_2_-induced increase in the Bax/Bcl-xL levels. *p < 0.05 vs the control group; # *p* < 0.05 vs the SiO_2_ group

Macrophages display marked phenotypic heterogeneity in response to environmental stimuli. In addition, macrophage activation plays a significant role in the pathogenesis of pulmonary fibrosis [47]. Generally, pulmonary fibrosis involves the initiation of inflammation caused by M1 macrophages, which can release various inflammatory mediators, followed by inflammation resolution, tissue repair, and fibrosis mediated by M2 macrophages. To further understand the functional relevance of SiO_2_-induced autophagy, we first measured the levels of phenotypic markers of macrophages, such as iNOS (M1), Arginase 1 (M2a) and SOCS3 (M2c) after exposure of U937 cells to SiO2. These markers were selected based on previous findings from our laboratory and have been considered as commonly accepted phenotypic markers of M1 and M2 macrophages [48, 49], As shown in Fig. 3a-b, SiO_2_ induced significant increases in the expression of iNOS, Arginase 1 and SOCS3. The expression of both the M1 and M2 markers peaked at 24 h and remained elevated until 48 h. Next, an autophagy inhibitor (3-methyladenine, 3-MA, 1 mM) was applied to determine the involvement of autophagy in macrophage activation. As shown in Fig. 3c-d, 3-MA significantly attenuated SiO_2_-induced macrophage activation, as indicated by decreased expression of iNOS, Arginase 1 and SOCS3.

### Autophagy was critical for macrophage apoptosis in response to silica

Given that mounting evidence suggests that macrophage death is a consequence of macrophage activation [50], we next measured cell viability after SiO_2_ exposure. As shown in Additional file 4: Figure S3 A-B, SiO_2_ induced a significant decrease in the viability of U937 cells, and this effect was attenuated by pretreatment with 3-MA. Moreover, apoptosis-related proteins such as Bax and cleaved caspase-3 were upregulated after SiO_2_ exposure, peaking at 24 h (Fig. 3e-h). The connection between autophagy and apoptosis involves numerous interactions that are multifaceted, complex and still poorly understood. Paradoxically, the pro-death and pro-survival functions of autophagy are cell- and context-dependent. [51–53] In our model, pretreatment with 3-MA attenuated SiO_2_-induced apoptosis, as indicated by decreases in the Bax/Bcl-xL and cleaved caspase-3 levels (Fig. 3i-j). These findings suggest a positive correlation between apoptosis and autophagy in the presence of SiO_2_.

### MCPIP1-mediated macrophage autophagy facilitated macrophage activation and apoptosis in U937 cell cultures

**Fig. 4:**
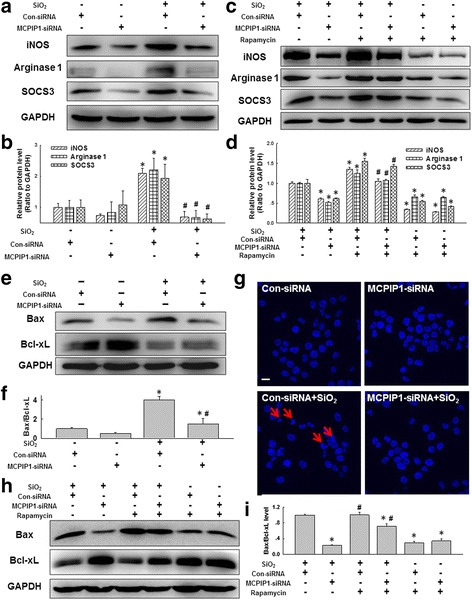
MCPIP1 induced macrophage apoptosis and fibrosis via autophagy. **a** Representative western blot showing the effects of RNAi targeting MCPIP1 on the expression of iNOS, Arginase 1 and SOCS3 induced by SiO_2_ treatment at 50 μg/cm^2^ for 24 h in U937 cells. **b** Densitometric analyses of five separate experiments suggested that RNAi targeting MCPIP1 attenuated the SiO_2_-induced increases in iNOS, Arginase 1 and SOCS3 expression. *p < 0.05 vs the corresponding control group; #*p* < 0.05 vs the corresponding SiO_2_ group. **c** Representative western blot showing the effects of rapamycin and RNAi targeting MCPIP1 on SiO_2_-induced expression of iNOS, Arginase 1 and SOCS3 in U937 cells. **d** Densitometric analyses of five separate experiments suggested that rapamycin reversed the effects of RNAi targeting MCPIP1 on SiO_2_-induced iNOS, Arginase 1 and SOCS3 expression. *p < 0.05 vs the corresponding SiO_2_ control group; #*p* < 0.05 vs the corresponding SiO_2_ + MCPIP1-siRNA group. **e** Representative western blot showing the effects of RNAi targeting MCPIP1 on SiO_2_-induced Bax and Bcl-xL expression in U937 cells. **f**. Densitometric analyses of five separate experiments suggested that RNAi targeting MCPIP1 attenuated the SiO_2_-induced increase in the Bax/Bcl-xL level. *p < 0.05 vs the control group; # *p* < 0.05 vs the SiO_2_ group. **g** Representative images of Hoechst 33342 staining demonstrating that SiO_2_-induced U937 cell apoptosis after SiO_2_ treatment for 24 h at 50 μg/cm^2^ was attenuated by RNAi targeting MCPIP1. Scale bar = 10 μm. **h** Representative western blot showing the effects of rapamycin and RNAi targeting MCPIP1 on the SiO_2_-induced expression of Bax and Bcl-xL in U937 cells. **i** Densitometric analyses of five separate experiments suggested that rapamycin reversed the effects of RNAi targeting MCPIP1 on the SiO_2_-induced increase in the Bax/Bcl-xL level. *p < 0.05 vs the SiO_2_ control group; #*p* < 0.05 vs the SiO_2_ + MCPIP1-siRNA group

After determining the role of autophagy in macrophage activation, it was reasonable to examine whether MCPIP1 induced macrophage activation via autophagy. First, RNAi targeting MCPIP1 was performed. As a result, the levels of macrophage activation markers after SiO_2_ exposure were significantly decreased (Fig. 4a-b). M1 macrophages characteristically possess filopodia that are more densely distributed than M2 macrophage filopodia. In contrast, the less densely distributed filopodia of M2 macrophages showed a flattened and elongated appearance [54]. We found that SiO_2_ exposure led to a changes in cell morphology towards M1 and M2 macrophages. However, knockdown of MCPIP1 markedly inhibited the effect of SiO_2_ treatment on macrophage differentiation (Additional file 5: Figure S4). Next, a specific activator of autophagy (rapamycin, 1 μM) was applied, and this treatment reversed the effect of RNAi targeting MCPIP1 on macrophage activation marker expression (Fig. 4c-d). Moreover, RNAi targeting MCPIP1 attenuated the SiO_2_-induced increase in the apoptosis index (Bax/Bcl-xL expression ratio) (Fig. 4e-f), and this inhibition of apoptosis was confirmed by Hoechst 33342 staining (Fig. 4g). Similarly, rapamycin treatment reversed the apoptosis-inhibiting effect of RNAi targeting MCPIP1 (Fig. 4h-i).

### SiO_2_-induced macrophage autophagy was involved in HPF-a cell activation and migration

**Fig. 5:**
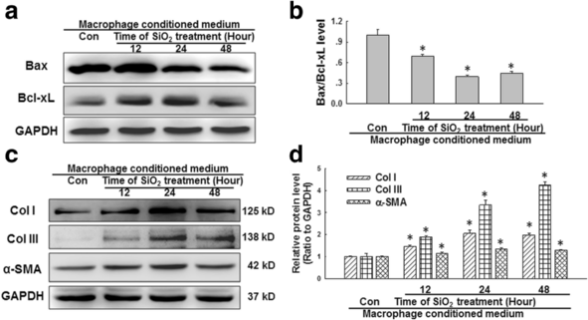
SiO_2_-induced macrophage autophagy was involved in HPF-a cell activation. **a** Representative western blot showing the effects of conditioned media from macrophages treated with SiO_2_ on Bax and Bcl-xL expression in HPF-a cells. **b** Densitometric analyses of five separate experiments suggested that the conditioned media from macrophages in the SiO_2_ group decreased the Bax/Bcl-xL level in HPF-a cells. *p < 0.05 vs the control group. **c**.Representative western blot showing the effects of conditioned media from macrophages on collagen I, collagen III and α-SMA expression in HPF-a cells. **d** Densitometric analyses of five separate experiments suggested that the conditioned media from macrophages in the SiO_2_ group increased the expression of collagen I, collagen III and α-SMA in HPF-a cells. *p < 0.05 vs the corresponding control group

Mounting evidence suggests that SiO_2_-induced macrophage activation initiates pulmonary fibrosis, characterized by fibroblast activation and collagen synthesis [55]. Therefore, we next explored whether MCPIP1-induced autophagy is also involved in changes in fibroblast functions. Supernatant from U937 cells subjected to SiO_2_ or vehicle treatments (conditioned medium) were applied to fibroblasts, and fibroblast proliferation and collagen synthesis were measured. As shown in Additional file 6: Figure S5A, the conditioned media from macrophages exposed to SiO_2_ for 12, 24 or 48 h promoted fibroblast proliferation after 24 h in culture. Moreover, the conditioned media induced fibroblast migration after 24 h in culture (Additional file 6: Figure S5B-C). Subsequently, the Bcl-xL and Bax levels in fibroblasts were measured. As shown in Fig. 5a-d, the conditioned media from macrophages exposed to SiO_2_ for 12, 24 or 48 h increased Bcl-xL, collagen I/III and α-SMA expression but decreased Bax expression in fibroblasts. These results indicated that apoptotic factors released by macrophages activated fibroblasts.

**Fig. 6:**
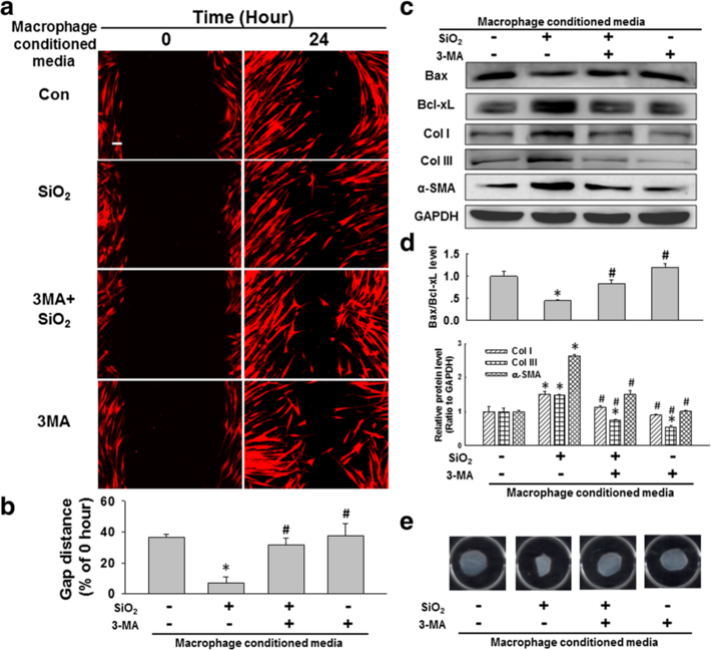
SiO_2_-induced macrophage autophagy was involved in HPF-a cell activation and migration. **a** Representative images showing the effects of the conditioned media from macrophages on the migration of RFP-labeled HPF-a cells. Scale bar = 80 μm. **b** Quantification of the scratch width in six separate experiments. *p < 0.05 vs the control group; #p < 0.05 vs the SiO_2_ group. **c** Representative western blot showing the effects of the conditioned media from macrophages on the expression of Bax, Bcl-xL, collagen I, collagen III and α–SMA in HPF-a cells. **d** Densitometric analyses of five separate experiments suggested that pretreatment of U937 macrophages with 3-MA attenuated the decrease in the Bax/Bcl-xL levels and the increases in collagen I, collagen III and α-SMA expression induced by the conditioned media from macrophages treated with SiO_2_. *p < 0.05 vs the control group; #*p* < 0.05 vs the SiO_2_ group. **e** Representative images of the collagen gel size showing the effects of the conditioned media from macrophages on gel contraction (indicating fibroblast activation)

To further understand the effect of SiO_2_-induced macrophage autophagy and apoptosis on changes in fibroblast functions, conditioned media from macrophages pretreated with 3-MA were applied to fibroblasts. As shown in Fig. 6a-b, the increase in fibroblast migration induced by SiO_2_-conditioned media was attenuated by pretreatment of U937 macrophages with 3-MA. Moreover, pretreatment of U937 macrophages with 3-MA also attenuated the increases in Bcl-xL, collagen I/III and α-SMA expression as well as the decrease in Bax expression in fibroblasts induced with SiO_2_-conditioned media (Fig. 6c-d). The results of the matrix contraction assay, a commonly employed assay to evaluate fibroblast activation, were shown in Fig. 6e. Pretreatment of U937 macrophages with 3-MA prevented gel contraction induced by SiO_2_-conditioned media. Furthermore, pretreatment of U937 macrophages with an apoptosis inhibitor (Z-VAD-FMK) exerted effects on fibroblast activation similar to those of 3-MA (Additional file 7: Figure S6 A-B).

These results indicated the possible existence of fibrosis-inducing factors released by macrophages activated fibroblasts. Next, the levels of the cytokines MCP-1, TNF-α and TGF-β, which have been established to play important roles in silicosis, were measured in macrophage supernatants. As shown in Additional file 8: Figure S7, SiO_2_ induced upregulation of MCP-1, TNF-α and TGF-β in U937 cells, and these changes were attenuated by pretreatment with 3-MA or Z-VAD-FMK.

### Knockdown of MCPIP1 in macrophages attenuated the pro-fibrogenic effects of conditioned media on fibroblasts

**Fig. 7:**
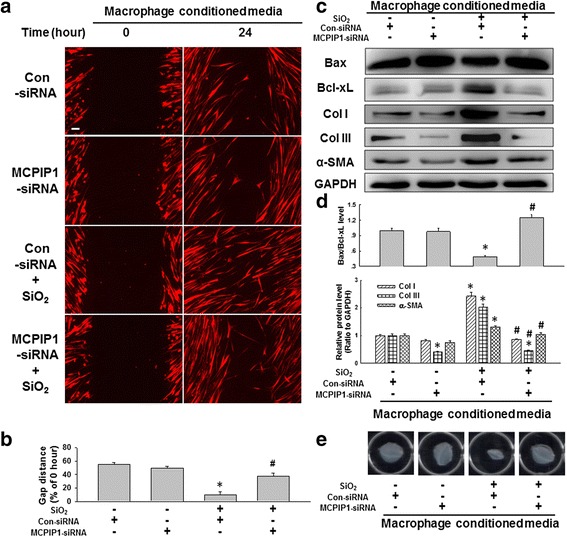
SiO_2_-induced MCPIP1 expression in macrophages was involved in HPF-a cell activation and migration. **a** Representative images showing the effects of conditioned media from macrophages on the migration of RFP-labeled HPF-a cells. Scale bar = 80 μm. **b** Quantification of the scratch width in six separate experiments. *p < 0.05 vs the control group; #*p* < 0.05 vs the SiO_2_ group. **c** Representative western blot showing the effects of conditioned media from U937 macrophages subjected to RNAi targeting MCPIP1 on Bax, Bcl-xL, collagen I, collagen III and α-SMA expression in HPF-a cells. **d** Densitometric analyses of five separate experiments suggested that RNAi targeting MCPIP1 attenuated the decrease in the Bax/Bcl-xL level and the increases in collagen I, collagen III and α-SMA expression induced by SiO_2_. *p < 0.05 vs the control group; #*p* < 0.05 vs the SiO_2_ group. **e** Representative images of the collagen gel size showing the effects of the conditioned media from macrophages on gel contraction

Having determined the role of SiO_2_-induced macrophage autophagy on changes in fibroblast functions, whether MCPIP1 is also involved in this process was investigated. As shown in Fig. 7a-b, the increase in fibroblast migration induced by SiO_2_-conditioned media was attenuated by RNAi targeting MCPIP1 in U937 macrophages. Moreover, RNAi targeting MCPIP1 in U937 macrophages attenuated the increases in Bcl-xL, collagen I/III and α-SMA expression as well as the decrease in Bax expression in fibroblasts induced with SiO_2_-conditioned media (Fig. 7c-d). Moreover, application of RNAi targeting MCPIP1 to U937 macrophages prevented gel contraction induced by SiO_2_-conditioned media (Fig. 7e). These results revealed the role of SiO_2_-induced MCPIP1 expression by macrophages in modulating the function of fibroblasts.

### Increased autophagy, apoptosis and activation were observed in macrophages from silicosis patients

**Fig. 8:**
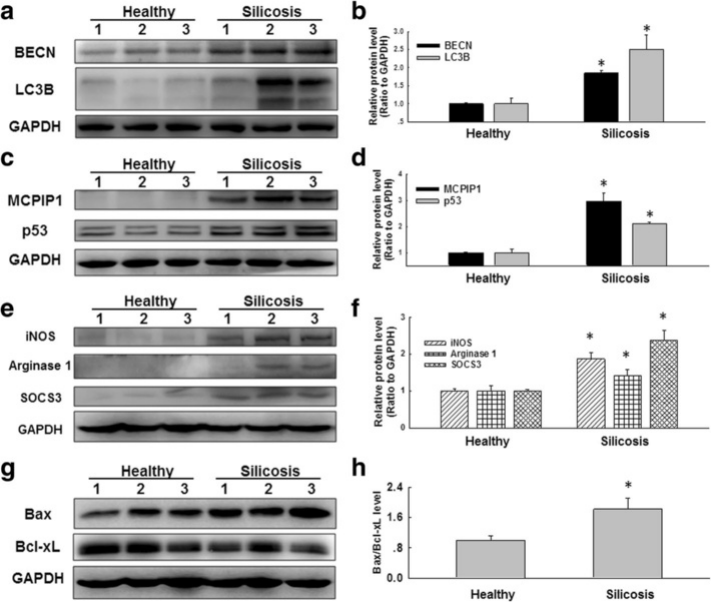
Autophagy was increased in macrophages from silicosis patients. **a** Representative western blot showing the expression of BECN and LC3B in macrophages from healthy donors and silicosis patients. **b** Densitometric analyses of macrophage samples from five healthy donors and five silicosis patients suggested that BECN and LC3B expression was elevated in macrophages from silicosis patients. *p < 0.05 vs the corresponding healthy control group. **c** Representative western blot showing the expression of MCPIP1 and p53 in macrophages from healthy donors and silicosis patients. **d** Densitometric analyses of macrophage samples from five healthy donors and five silicosis patients suggested that MCPIP1 and p53 expression was elevated in macrophages from silicosis patients. *p < 0.05 vs the corresponding healthy control group. **e** Representative western blot showing the expression of iNOS, Arginase 1 and SOCS3 in macrophages from healthy donors and silicosis patients. **f** Densitometric analyses of macrophage samples from five healthy donors and five silicosis patients suggested that iNOS, Arginase 1 and SOCS3 expression was increased in macrophages from silicosis patients. *p < 0.05 vs the corresponding healthy control group. **g** Representative western blot showing the expression of Bax and Bcl-xL in macrophages from healthy donors and silicosis patients. **h** Densitometric analyses of macrophage samples from five healthy donors and five silicosis patients suggested that the Bax/Bcl-xL level was elevated in macrophages from silicosis patients. *p < 0.05 vs the healthy control group

To validate our findings, we next extended our cell culture experiments to the examination of silicosis patients. As shown in Fig. 8a-b, the LC3B and BECN levels were significantly increased in macrophages from BALF of silicosis patients compared with that of healthy donors. p53 can induce and regulate autophagy through its transactivation function in a context-dependent manner [46]. In addition, p53-induced autophagy contributes to the occurrence of apoptosis. Thus, we measured the expression of p53 as well as MCPIP1 in macrophages from human BALF. As shown in Fig. 8c-d, both the MCPIP1 and p53 levels were significantly increased in macrophages from BALF of silicosis patients. Furthermore, macrophage activation and apoptosis marker expression were increased in macrophages from BALF of silicosis patients (Fig. 8e-h). Taken together, these results indicated that macrophages from silicosis patients showed increased autophagy, apoptosis and activation, which promoted the development of silicosis.

## Discussion

Silicosis is a disorder that is characterized by abnormalities in collagen synthesis and deposition within the lung [56, 57]. The pathogenesis of silicosis, for which there is still no effective clinical therapy, remains unknown. Autophagy, an important physiological regulatory factor, plays a central role in many lung pathologies, including IPF and cystic fibrosis [20]. Recently, considerable evidence has indicated that nanoparticles endocytosed by a variety of cells can cause autophagosome formation and that dysfunction of autophagy is involved in the toxicity of nanoparticles. Wan et al. [58] found that carbon nanomaterials induced autophagosome accumulation but inhibited the degradation of the autophagic substrate p62. Their further investigations indicated that accumulation of carbon nanomaterials in macrophage lysosomes resulted in impaired lysosome function, which strongly decreased autophagic degradation. This process was shown to be a potential mechanism of the toxic effects of nanomaterials on cells. These findings suggest that autophagic defects may mediate nanomaterial-induced cell damage. Other studies [59] also showed that autophagy was required for airway epithelial injury induced by ultrafine particulate matter, which consists of larger particles (0.1 ~ 0.5 μm) than nanoparticles and which causes airway inflammation and mucus hyperproduction.

Recent studies have demonstrated that autophagy is involved in the pathogenesis of silicosis [26]. However, the molecular mechanisms underlying the activation and progression of autophagy in silicosis are not fully understood. Because human leukemic U937 cells have been widely used to study the differentiation of premonocytes into monocyte-like cells [60], in this study, PMA-treated U937 cells that had differentiated towards adherent macrophage-like cells were used to study the effects of SiO_2_ on human macrophages. In several professions, including metal mining, tunnel excavation and cement manufacturing, long-term exposure to silica dust leads to silicosis. Silicosis develops slowly following dust exposure for 5–10 years or as long as 15–20 years in some cases. The development, progression and severity of silicosis have been associated with the cumulative level of silica dust exposure to the lungs. There is rarely only one component of silica in a production environment; instead, a variety of components of silica are often present simultaneously. Therefore, the complicated pathogenesis of silicosis involves the combined effect of a mixture of mineral dusts. Studies [2, 61] suggested that silica caused alveolar macrophage (AM) death during the process of silicotic fibrosis, which indicated that AM death plays a critical role in the development of silicosis. Macrophages exposed to 50 μg/cm^2^ silica for 24 h showed a significantly increased apoptotic rate to approximately 30 %; this value is comparable to that observed in a study of a mouse macrophage cell line (MH-S cells), in which silica exposure for 6 h at the same dose resulted in an apoptotic rate of ~15 %. We found that autophagy was significantly increased in macrophages that were differentiated from U937 cells in our in vitro model of silica exposure. This observation demonstrated that autophagy in macrophages may be critical for silicosis. Many studies have shown that MCPIP1 promotes autophagy, which participates in the development of a variety of diseases; thus, we examined whether MCPIP1 also mediates macrophage autophagy in an in vitro model of silica exposure. First, we treated macrophages with SiO_2_ and found significantly increased MCPIP1 levels. Immunofluorescence analysis showed increased MCPIP1 expression accompanied by enhanced LC3B expression. Moreover, siRNA-mediated depletion of MCPIP1 significantly attenuated autophagic activity, as indicated by the decreased protein expression of LC3B and BECN and decreased levels of autophagosomes and autophagolysosomes. The present data suggest that MCPIP1 is involved in macrophage autophagy in our in vitro model of silica exposure. Nanoparticles, which are less than 100 nm, can be phagocytosed into multiple cells, leading to lysosomal damage and dysfunction as well as autophagic blockade. However, the silica used in our study was less than 5 μm, far larger than nanoparticles. In this study, treatment of macrophages with silica promoted autophagic flux rather than decreasing autophagy, which is the effect of nanomaterials on cells. The distinct effects of particles of different sizes may be supported by existing evidence.

Alveolar macrophages are the most prevalent resident cells in the lung. Persistent exposure to silica mediates SiO_2_-induced responses, including the activation and apoptosis of alveolar macrophages. Macrophages exhibit significant heterogeneity and plasticity of function and phenotype in response to external stimuli in the environment. From previous studies, it was known that activated macrophages primarily polarize towards the M1 and M2 phenotypes and play various roles in physiological and pathological processes. Generally, cell death could be categorized as programmed cell death (PCD) or necrosis [62]. Necrosis is a mode of abnormal death that occurs in response to external stimuli. In contrast to the indicators of apoptosis, the morphological features of necrosis are often accompanied by inflammation. Apoptotic signaling stimulates downstream signaling through membrane receptor pathways, activates caspase-3, and then initiates apoptosis [63, 64]. In addition, apoptotic PCD can occur via a caspase-independent mechanism. Autophagic cell death was also known as type II PCD, in which numerous vacuoles appear in the cytoplasm surrounding cytoplasmic proteins and organelles and the content within vacuoles is degraded. The question remains as to whether autophagy triggers macrophage activation and apoptosis, thus leading to fibrosis. In the present study, we found that SiO_2_ significantly upregulated markers of both M1 and M2 macrophages: iNOS (M1), Arginase 1 and SOCS3 (M2). Morphologically, SiO_2_ promoted the conversion of macrophages towards the M1 and M2 phenotypes. In addition, apoptosis-related protein expression was also significantly increased following SiO_2_ exposure based on western blot assays. Other reports conflict with our results regarding macrophage activation in response to external stimulatory factors. There is generally an inverse correlation between the M1 and M2 phenotypes of macrophages exposed to environmental stimuli. However, we demonstrated here that both M1 and M2 macrophage markers were upregulated in response to SiO_2_ in a time-dependent manner. According to previous reports, PMA-treated monocytes differentiate into M0 macrophages, which differentiate into activated M1 or M2 macrophages upon incubation with different stimuli, such as LPS and IFN-γ or IL-4 and IL-13, respectively [9, 65, 66]. Therefore, it is possible that in this study, U937 cells stimulated with PMA differentiated into M0 macrophages, which were further induced to express M1 and M2 markers after SiO_2_ treatment, as we demonstrated. It was speculated that following exposure to SiO_2_, two distinct groups of macrophages differentiated from M0 macrophages induced via PMA treatment [54], and this possibility warrants further investigation. These data suggest that autophagy promotes macrophage activation and apoptosis in our model of silica exposure.

MCPIP1 plays a role in regulating autophagy and subsequently promoting cell apoptosis [67]. In this study, we observed that MCPIP1 was critical for autophagy in our model of silica exposure. Moreover, MCPIP1 facilitated the apoptosis and activation of macrophages by mediating autophagy. However, previous studies [68] highlighted the presence of considerable inflammatory cell infiltration into other organs, especially the lungs and the liver, concurrent with massive inflammatory factor secretion in MCPIP1-deficient mice, which was significantly ameliorated by antibiotic administration. Those findings suggested that MCPIP1 possessed marked anti-inflammatory effects. In contrast, MCPIP1 expression has been found to induce adipogenesis and osteoclast differentiation via oxidative stress and endoplasmic reticulum stress [44, 69]; these findings indicate a notable proinflammatory role of MCPIP1. These previous results led us to speculate that MCPIP1, as a novel regulator, might prevent inflammation and maintain body function under normal conditions. Thus, aberrant MCPIP1 expression, whether increased or reduced, can result in various abnormalities in cellular function via deubiquitinase activity [70] RNase activity [35] or transcriptional activity [44], depending on the external environmental conditions.

p53, a tumor suppressor, is an important regulator of cell apoptosis and the intracellular environment [71]. Recent reports have revealed that nuclear rather than cytoplasmic p53 can also promote autophagy and that the functions of p53 depend on the nature and extent of the stress induced, which can lead to different biological outcomes [72]. Interestingly, our study showed that p53 expression was significantly increased in macrophages in response to SiO_2_ (results not shown). Further examination indicated that p53 maintained the effects of MCPIP1 in the regulation of autophagy. In addition, MCPIP1 may regulate autophagy by directly interacting with p53. Together with these observations, our findings indicate that MCPIP1 mediates macrophage autophagy, apoptosis and activation via p53 activation in silicosis models.

There is evidence that in the pulmonary interstitium, large amounts of macrophages release various cytokines, such as TGF-β, alveolar macrophage-derived growth factor (AMDGF) and fibronectin, and can stimulate fibroblast proliferation as well as collagen synthesis and deposition [73, 74]. In this study, we found that fibroblasts incubated in conditioned media from macrophages that were exposed to SiO_2_ for 24 h showed enhanced migration, proliferation, activation and collagen synthesis compared to fibroblasts incubated in media from untreated macrophages. These observations suggest the existence of cytokines from macrophages that activate fibroblasts, as verified in our study. Evaluation of cytokine levels via ELISA revealed that SiO_2_ increased the production of TGF-β, MCP-1 and TNF-α but that the levels of these cytokines were significantly decreased after 3-MA or Z-VAD-FMK treatment. Furthermore, inhibiting autophagy and apoptosis in macrophages reduced fibroblast activation caused by incubation in conditioned media from macrophages in the in vitro model of silica exposure. However, enhancement of macrophage autophagy further increased the pro-fibrogenic stimulatory effect of the conditioned media from macrophages. Our data indicate that macrophages act as paracrine effectors to modulate fibroblast proliferation and migration and that macrophage autophagy plays a central role in these effects. Studies in recent years showed a complicated relationship between autophagy and apoptosis [75]. These processes were mutually inducing in some cases but antagonizing in other cases. Autophagy and apoptosis can occur within a cell successively or even simultaneously, leading to cell death. In our research, silica caused macrophage autophagy and apoptosis, which promoted fibrosis; however, inhibiting apoptosis significantly reduced the development of fibrosis. Inhibiting autophagy using 3-MA also alleviated the decrease in cell viability, suppressed apoptosis, and reduced pulmonary fibrosis caused by SiO_2_ exposure. These data suggest that both autophagy and apoptosis play crucial roles in the development of silicosis and that they could induce one other in our experimental model. Our results also demonstrated that MCPIP1 promoted the autophagic process in macrophages in response to silica exposure. Moreover, MCPIP1 knockdown in macrophages dramatically decreased the pro-fibrogenic stimulatory effect of conditioned media from macrophages that were exposed to SiO_2_. These findings indicate that MCPIP1 mediates autophagy through p53 signaling and is involved in the pro-fibrogenic effects of conditioned media from macrophages in our in vitro model of silica exposure.

In addition, our analyses of primary alveolar macrophages from patients suggested that the expression levels of MCPIP1, p53 and autophagic proteins were increased in association with enhanced macrophage apoptosis and activation compared to primary alveolar macrophages from healthy donors, in agreement with our in vitro results. Thus, our in vivo and in vitro results confirmed the clinical significance of our findings and demonstrated MCPIP1-mediated autophagy may serve as a potential marker of silicosis.

## Conclusion

**Fig. 9:**
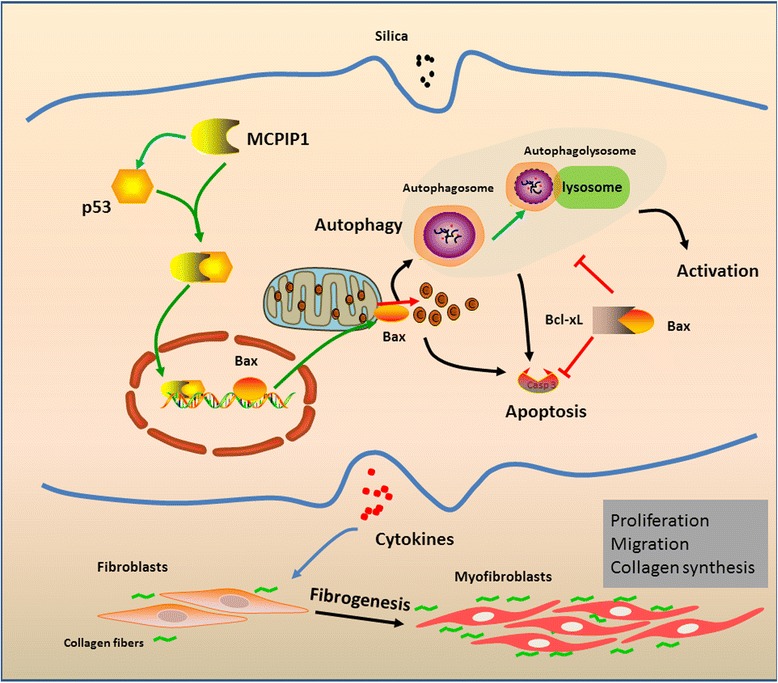
Schematic diagram showing the mechanisms by which MCPIP1 in macrophages mediates silica-induced pulmonary fibrosis. MCPIP1 expression was increased in macrophages exposed to SiO2, leading to the subsequent enhancement of p53 expression. The interaction between MCPIP1 and p53 may be involved in the regulation of gene transcription and may enhance Bax expression, thereby promoting autophagic and apoptotic processes. Enhanced autophagy further stimulated macrophage apoptosis and activation, which resulted in overproduction of pro-fibrogenic cytokines by macrophages. Fibroblasts responding to these cytokines differentiated into myofibroblasts, which showed enhanced proliferation and migration capacities as well as increased collagen synthesis

MCPIP1 induces autophagy through p53 signaling in macrophages exposed to SiO_2_, leading to macrophage apoptosis and activation, and regulates pulmonary fibroblast proliferation and migration (Fig. 9). Our results suggest that MCPIP1/p53-mediated macrophage autophagy plays an important role in the development of silicosis and that targeting MCPIP1/p53-mediated autophagic signaling represents a promising therapeutic strategy to prevent silicosis.

## Additional files

Additional file 1: Table S1. Characterization of silica particles in the culture medium. Microscopic images of the dispersion state of silica particles in the culture medium in the presence of cells. Scale bar = 200 µm.
Additional file 2: Figure S1. Dose-response effect of SiO_2_ on BECN and LC3B expression. **A**. Representative western blot showing the effects of different dosages of SiO_2_ on the expression of BECN and LC3B in U937 cells at 24 h after SiO_2_ treatment. Densitometric analyses of five separate experiments suggested that SiO_2_ induced BECN (**B**) and LC3B (**C**) expression in a dose-dependent manner. * *p* < 0.05 vs the control group; # *p* < 0.05 vs the SiO_2_ group.
Additional file 3: Figure S2. Effect of SiO_2_ on MCPIP1 and p53 expression in U937 cells. Representative immunocytochemical images showing that SiO_2_ (50 µg/cm^2^) increased the expression of MCPIP1 and p53 in U937 cells at 24 h following SiO_2_ treatment. Scale bar = 20 μm.
Additional file 4: Figure S3. Effect of SiO_2_ on U937 cell viability. **A**. MTT assay showing a time-dependent decrease in U937 cell viability induced by 50 µg/cm^2^ SiO_2_. * *p* < 0.05 vs the 0-hour group. **B**. MTT assay showing that 3-MA abolished the decrease in cell viability induced by SiO_2_ treatment for 24 h. * *p* < 0.05 vs the control group; # *p* < 0.05 vs the SiO_2_ group.
Additional file 5: Figure S4. Representative images showing the effects of RNAi targeting MCPIP1 on the changes in macrophage morphology induced by SiO_2_ treatment at 50 µg/cm^2^ for 24 h in U937 cells. SiO_2_ exposure led to morphologic changes in macrophages towards the M1 (arrow) and M2 phenotypes (arrowhead). However, knockdown of MCPIP1 significantly alleviated this effect of SiO_2_ treatment on macrophages (Figure S4). Scale bar = 200 µm.
Additional file 6: Figure S5. Effects of conditioned medium from macrophages on HPF-a cell activation and migration. **A**. MTT assay showing that the conditioned medium from macrophages altered HPF-a cell viability. * *p* < 0.05 vs the control group. **B**. Representative images showing the effects of conditioned medium from macrophages exposed to SiO_2_ or sterile saline for 24 h on migration by RFP-labeled HPF-a cells. Scale bar = 80 µm. **C**. Quantification of the scratch width in six separate experiments. * *p* < 0.05 vs the control group.
Additional file 7: Figure S6. Effects of macrophage apoptosis on HPF-a cell activation and migration. **A**. Conditioned medium was obtained from macrophages incubated in the presence or absence of Z-VAD-FMK for 1 h prior to treatment with SiO_2_, and this medium was applied to cultures of HPF-a cells for 24 h. Representative western blot showing the effects of conditioned medium from macrophages on the expression of collagen I, collagen III and α–SMA in HPF-a cells. **B**. Densitometric analyses of five separate experiments suggested that pretreatment of macrophages with Z-VAD-FMK attenuated the increases in collagen I, collagen III and α-SMA expression levels in fibroblasts induced by application of conditioned medium from macrophages that were treated with SiO_2_. * *p* < 0.05 vs the control group; # *p* < 0.05 vs the SiO_2_ group.
Additional file 8: Figure S7. Effects of SiO_2_ on the levels of macrophage-derived cytokines. ELISAs suggested that the upregulated expression of MCP-1, TNF-α and TGF-β in macrophages induced by SiO_2_ treatment for 24 h was attenuated by pretreatment with 3-MA or Z-VAD-FMK. * *p* < 0.05 vs the corresponding control group; # *p* < 0.05 vs the corresponding SiO_2_ group.

## Abbreviations

3-MA: 3-methyladenine
AMDGF: Alveolar macrophage-derived growth factor
BALF: Bronchoalveolar lavage fluid
CCL2: C-C chemokine ligand 2
CCR2: C-C chemokine receptor 2
COPD: Chronic obstructive pulmonary disease
DMEM: Dulbecco's modified eagle's medium
FBS: Fetal bovine serum
HPF-a: Human pulmonary fibroblasts-adult
iNOS: Inducible nitric oxide synthase
IPF: Idiopathic pulmonary fibrosis
MCP-1: Monocyte chemotactic protein-1
MCPIP1: Monocyte chemotactic protein-1-induced protein 1
MTT: 3-(4,5-dimethylthiazol-2-yl)-2,5-diphenyltetrazolium bromide
NGS: Normal goat serum
NS: Normal saline
PAH: Pulmonary arterial hypertension
PMA: Phorbol myristate acetate
SiO_2_: Silicon dioxide
siRNA: Small interference RNA
SOCS3: Suppressor of cytokine signaling 3
α-SMA: α smooth muscle actin
