# Supplementary material for: Macrophage-derived MCPIP1 mediates silica-induced pulmonary fibrosis via autophagy
Source: Part Fibre Toxicol. 2016 Oct 25;13:55. doi: 10.1186/s12989-016-0167-z (PMC5078901; doi:10.1186/s12989-016-0167-z)
Supplement: Additional file 1: Table S1. — Characterization of silica particles in the culture medium. Microscopic images of the dispersion state of silica particles in the culture medium in the presence of cells. Scale bar = 200 µm. [file 12989_2016_167_MOESM1_ESM.doc]

**Supplementary Information**

Manuscript title: Macrophage-derived MCPIP1 mediates silica-induced pulmonary fibrosis via autophagy

Authors: Liu, Fang, Wang, Cheng, Zhang, Liao, Yao, Chao

**Table of Contents**

Supplementary Table S1 2

Supplementary Figure S1 3

Supplementary Figure S2 4

Supplementary Figure S3 5

Supplementary Figure S4 6

Supplementary Figure S5 7

Supplementary Figure S6 8

**Table 1 Characterization of silica particles in the culture medium**

| Characteristics | Silica particles |
| --- | --- |
| Size and distribution | 1-5 μm (> 80%) |
| Deposition rate | 5 min (> 95%) |
| Aggregation^[a]^ | - |
| Crystalline structure | Microcrystalline |

^[a]^ (-) no aggregation, (+) aggregation


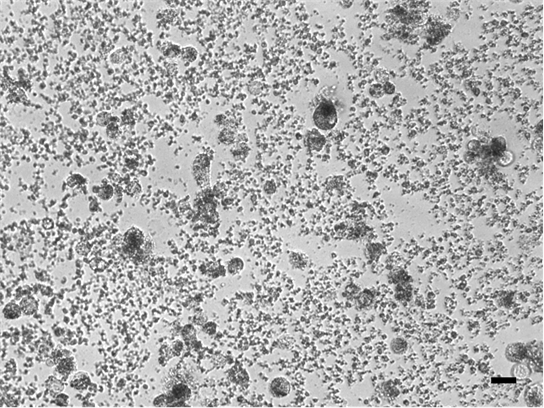


Microscopic images of the dispersion state of silica particles in the culture medium in the presence of cells. Scale bar=200 µm.

Supplementary Figure S1


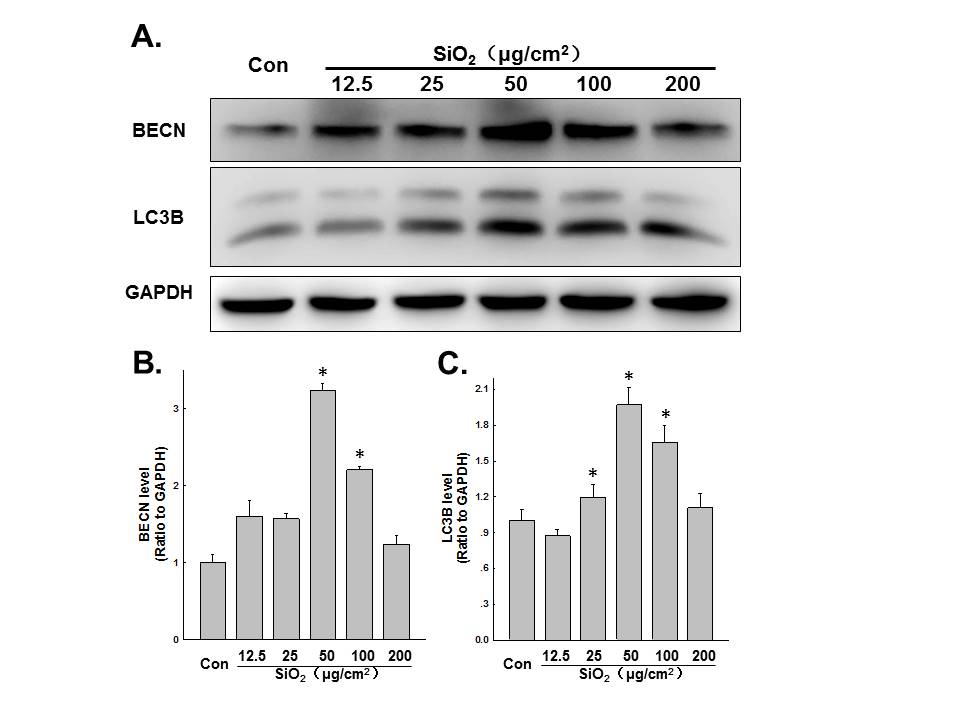


**Fig. S1** Dose-response effect of SiO_2_ on BECN and LC3B expression.

**A.** Representative western blot showing the effects of different dosages of SiO_2_ on the expression of BECN and LC3B in U937 cells at 24 h after SiO_2_ treatment. Densitometric analyses of five separate experiments suggested that SiO_2_ induced BECN **(B)** and LC3B **(C)** expression in a dose-dependent manner. * p<0.05 vs the control group; # p<0.05 vs the SiO_2_ group.

Supplementary Figure S2


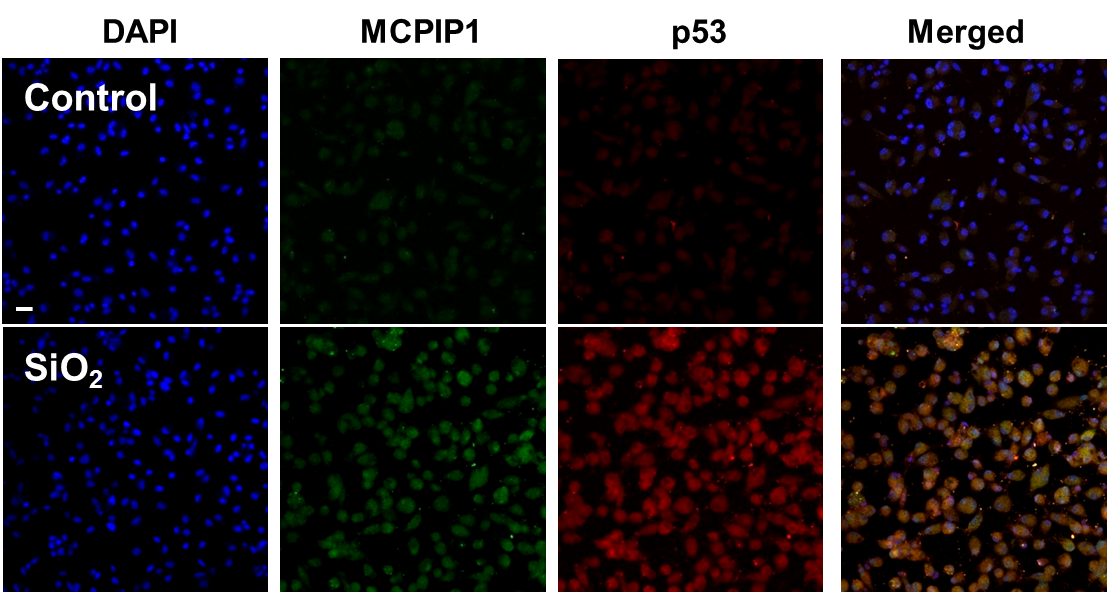


**Fig. S2** Effect of SiO_2_ on MCPIP1 and p53 expression in U937 cells

Representative immunocytochemical images showing that SiO_2_ (50 µg/cm^2^) increased the expression of MCPIP1 and p53 in U937 cells at 24 h following SiO_2_ treatment. Scale bar=20 μm.

Supplementary Figure S3


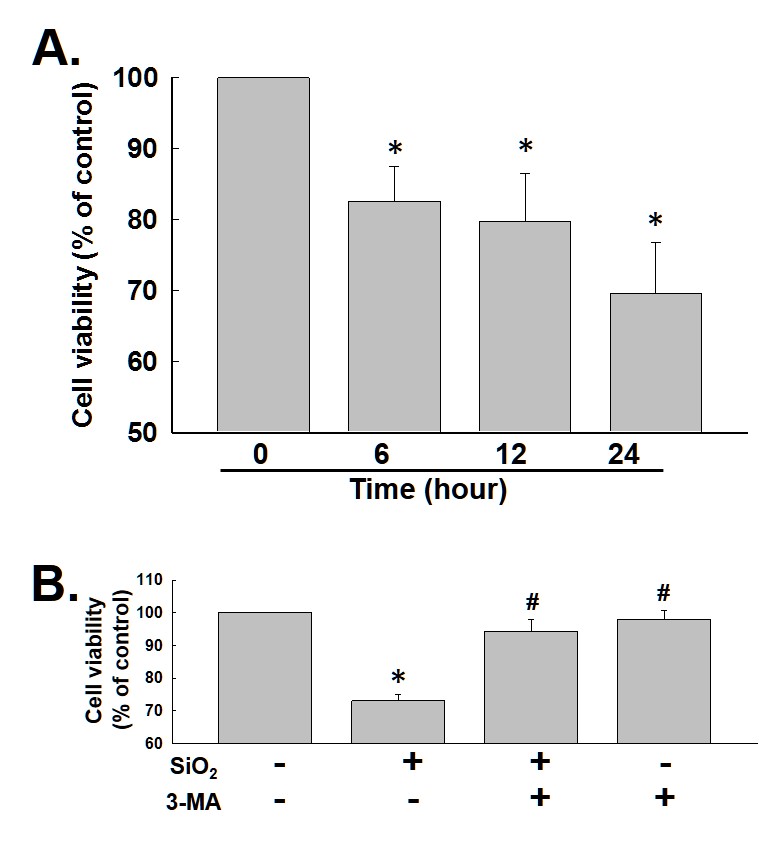


**Fig. S3** Effect of SiO_2_ on U937 cell viability.

**A.** MTT assay showing a time-dependent decrease in U937 cell viability induced by 50 µg/cm^2^ SiO_2_. * p<0.05 vs the 0-hour group. **B.** MTT assay showing that 3-MA abolished the decrease in cell viability induced by SiO_2_ treatment for 24 h. * p<0.05 vs the control group; # p<0.05 vs the SiO_2_ group.

Supplementary Figure S4


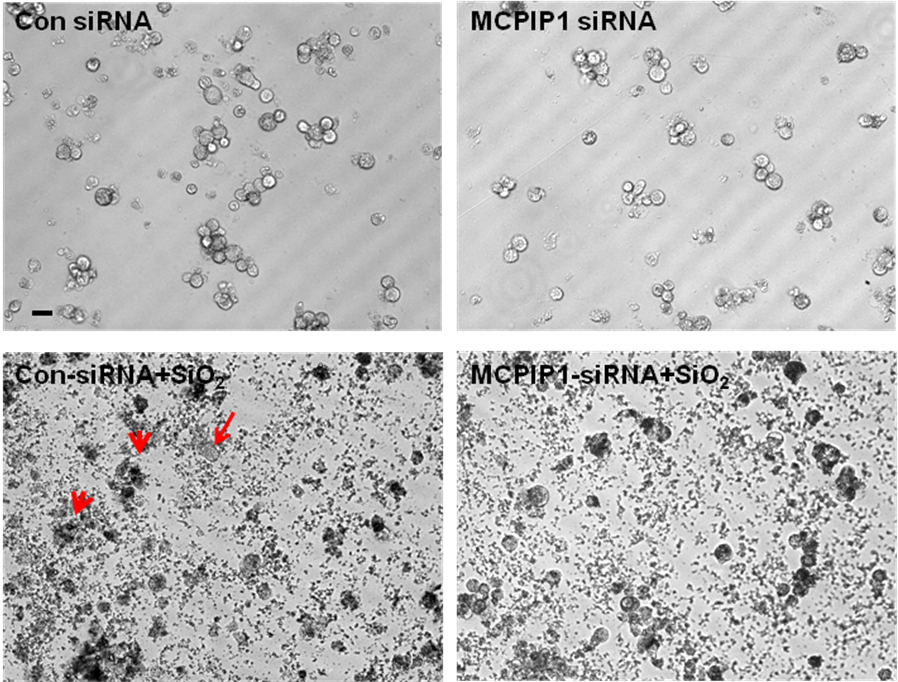


**Fig. S4** Representative images showing the effects of RNAi targeting MCPIP1 on the changes in macrophage morphology induced by SiO_2_ treatment at 50 µg/cm^2^ for 24 h in U937 cells. SiO_2_ exposure led to morphologic changes in macrophages towards the M1 (arrow) and M2 phenotypes (arrowhead). However, knockdown of MCPIP1 significantly alleviated this effect of SiO_2_ treatment on macrophages (Figure S4). Scale bar=200 µm.

Supplementary Figure S5


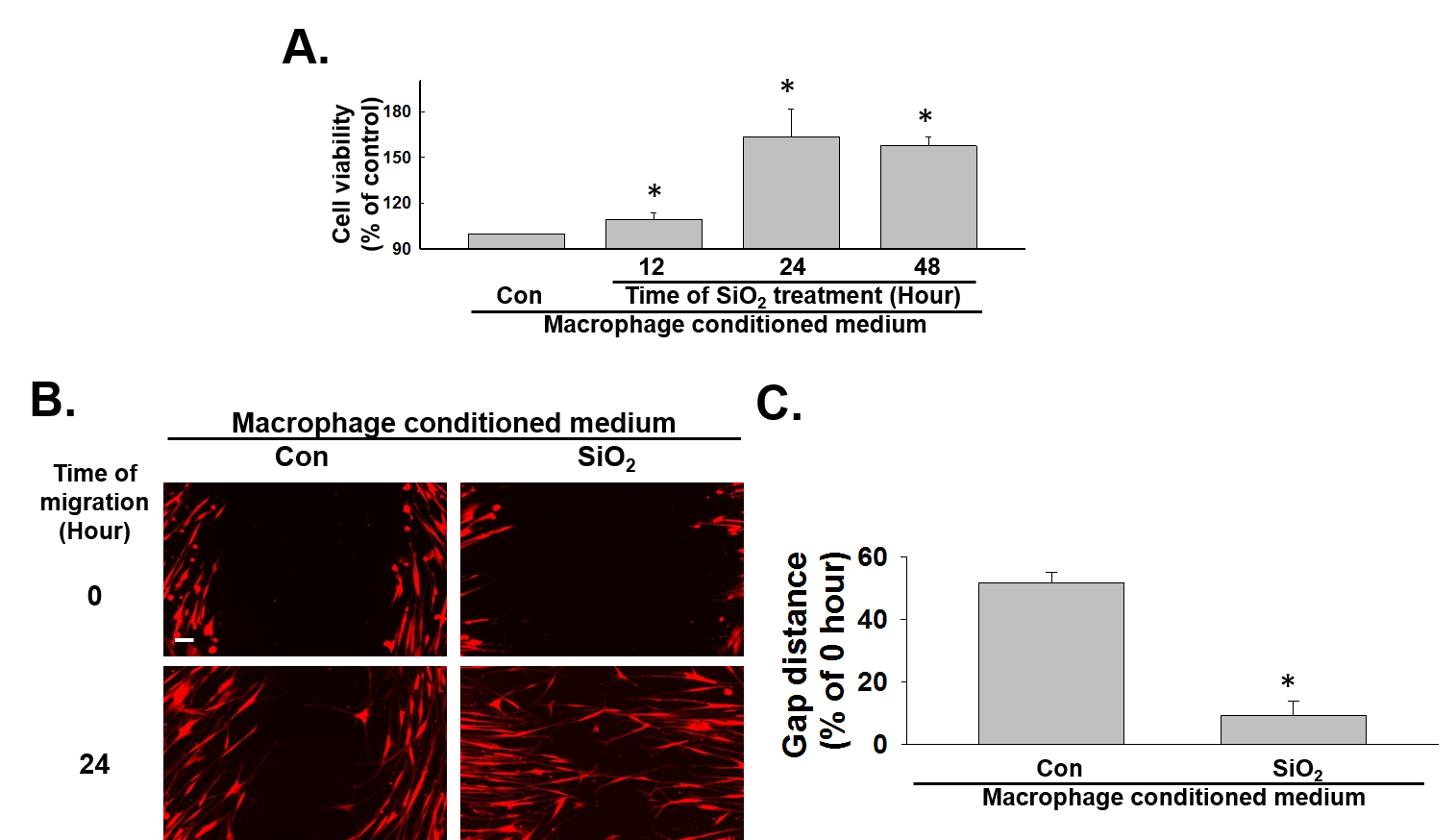


**Fig. S5** Effects of conditioned medium from macrophages on HPF-a cell activation and migration.

**A.** MTT assay showing that the conditioned medium from macrophages altered HPF-a cell viability. * p<0.05 vs the control group. **B.** Representative images showing the effects of conditioned medium from macrophages exposed to SiO_2_ or sterile saline for 24 h on migration by RFP-labeled HPF-a cells. Scale bar=80 µm. **C.** Quantification of the scratch width in six separate experiments. * p<0.05 vs the control group.

Supplementary Figure S6


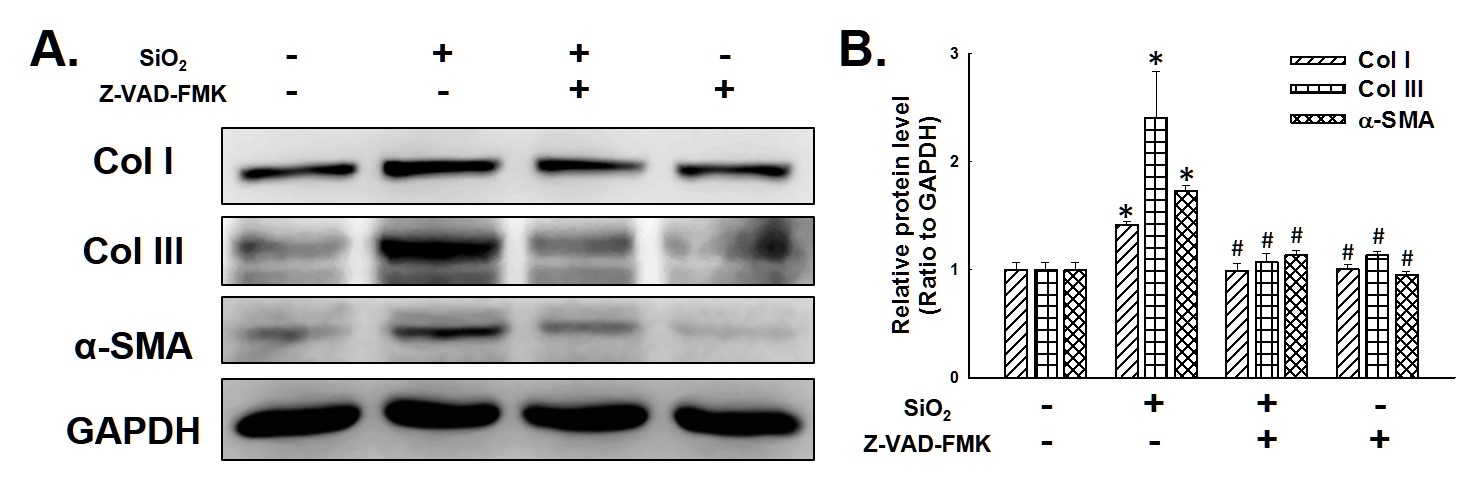


**Fig. S6** Effects of macrophage apoptosis on HPF-a cell activation and migration.

**A.** Conditioned medium was obtained from macrophages incubated in the presence or absence of Z-VAD-FMK for 1 h prior to treatment with SiO_2_, and this medium was applied to cultures of HPF-a cells for 24 h. Representative western blot showing the effects of conditioned medium from macrophages on the expression of collagen I, collagen III and α–SMA in HPF-a cells. **B.** Densitometric analyses of five separate experiments suggested that pretreatment of macrophages with Z-VAD-FMK attenuated the increases in collagen I, collagen III and α-SMA expression levels in fibroblasts induced by application of conditioned medium from macrophages that were treated with SiO_2_. * p<0.05 vs the control group; # p<0.05 vs the SiO_2_ group.

Supplementary Figure S7


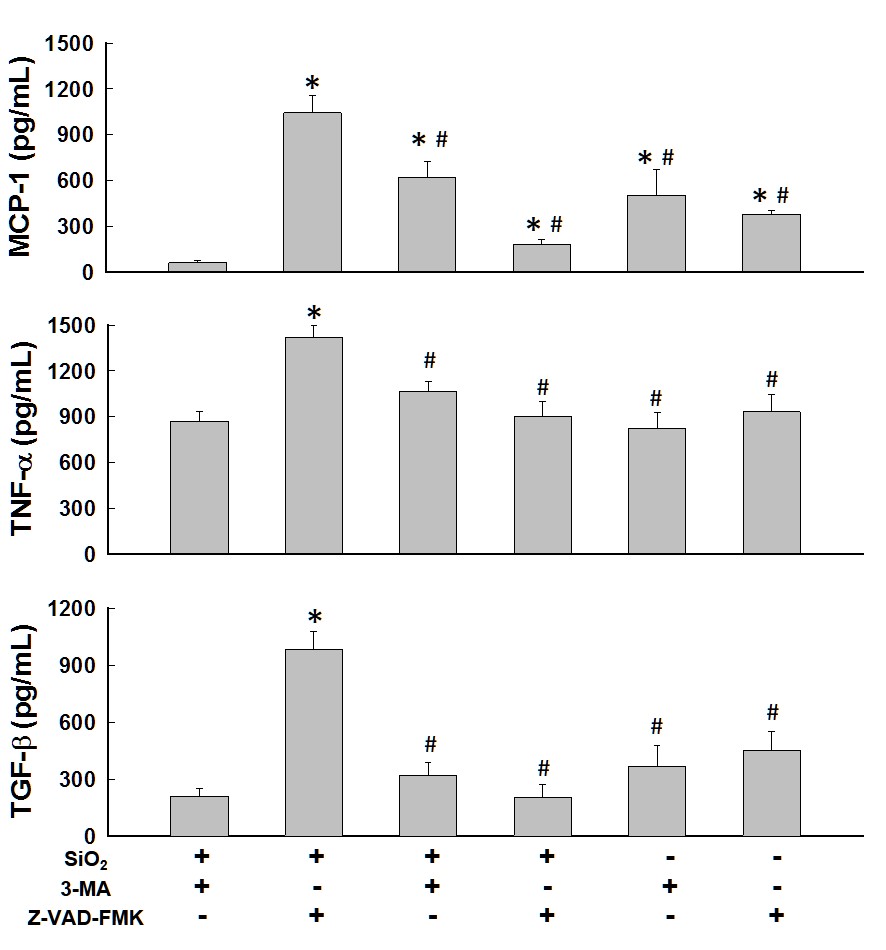


**Fig. S7** Effects of SiO_2_ on the levels of macrophage-derived cytokines.

ELISAs suggested that the upregulated expression of MCP-1, TNF-α and TGF-β in macrophages induced by SiO_2_ treatment for 24 h was attenuated by pretreatment with 3-MA or Z-VAD-FMK. * p<0.05 vs the corresponding control group; # p<0.05 vs the corresponding SiO_2_ group.
