# Supplementary figures and images for: Macrophage-derived MCPIP1 mediates silica-induced pulmonary fibrosis via autophagy
Source: Part Fibre Toxicol. 2016 Oct 25;13:55. doi: 10.1186/s12989-016-0167-z (PMC5078901; doi:10.1186/s12989-016-0167-z)

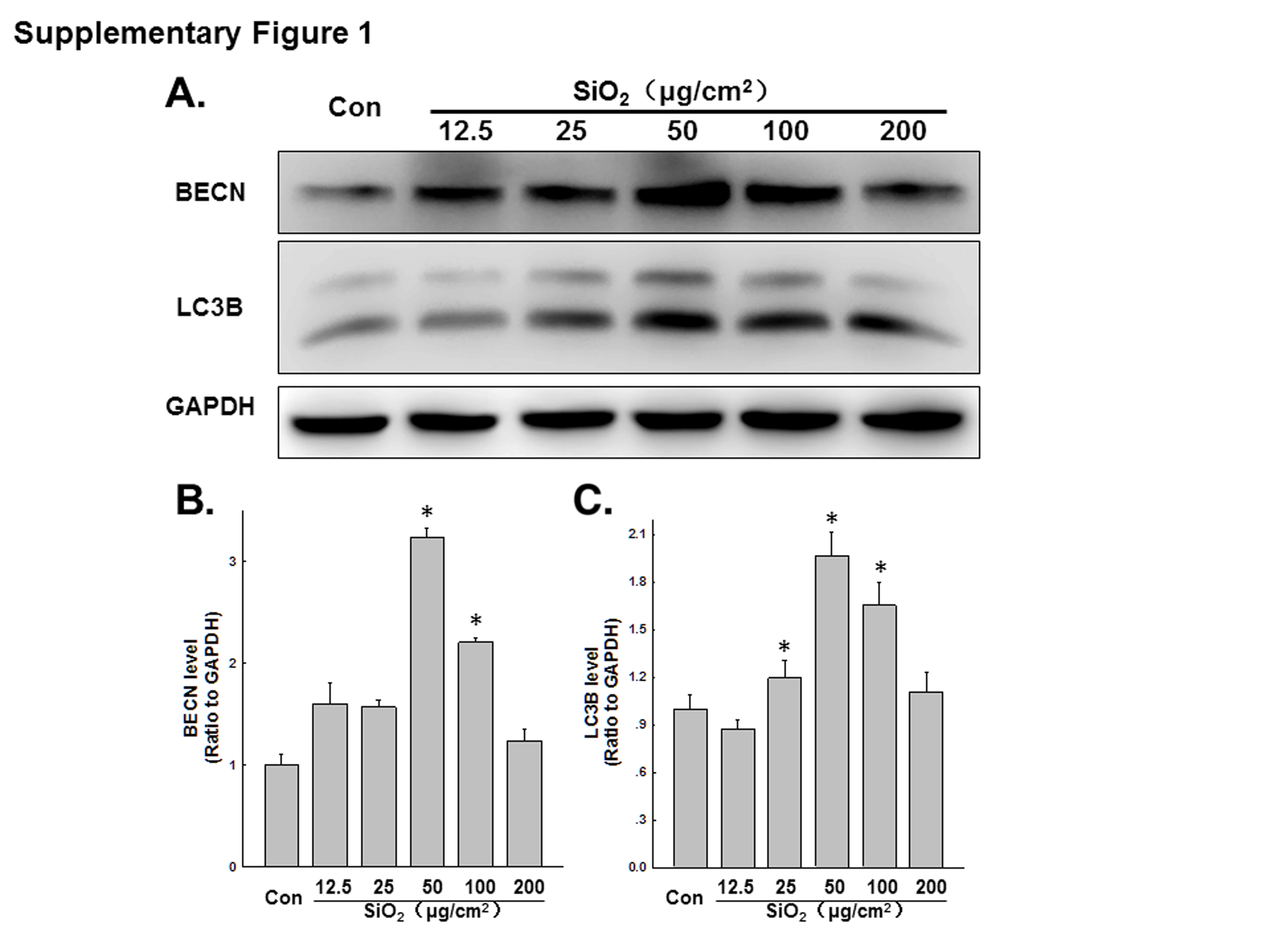

Supplement: Additional file 2: Figure S1. — Dose-response effect of SiO2 on BECN and LC3B expression. A. Representative western blot showing the effects of different dosages of SiO2 on the expression of BECN and LC3B in U937 cells at 24 h after SiO2 treatment. Densitometric analyses of five separate experiments suggested that SiO2 induced BECN (B) and LC3B (C) expression in a dose-dependent manner. * p < 0.05 vs the control group; # p < 0.05 vs the SiO2 group. [file 12989_2016_167_MOESM2_ESM.tif]

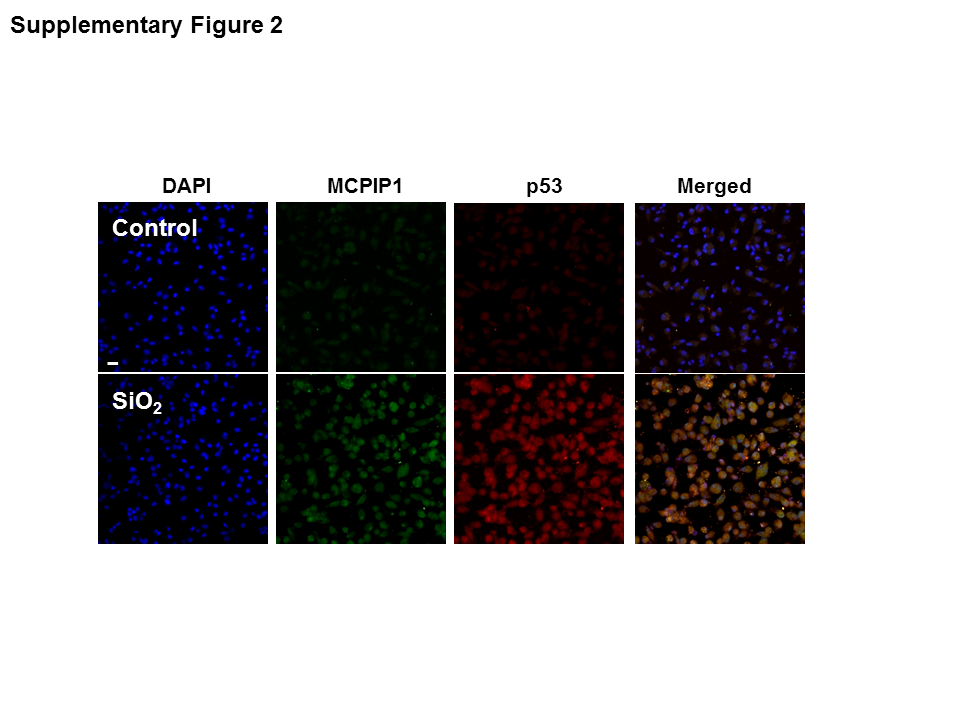

Supplement: Additional file 3: Figure S2. — Effect of SiO2 on MCPIP1 and p53 expression in U937 cells. Representative immunocytochemical images showing that SiO2 (50 µg/cm2) increased the expression of MCPIP1 and p53 in U937 cells at 24 h following SiO2 treatment. Scale bar = 20 μm. [file 12989_2016_167_MOESM3_ESM.tif]

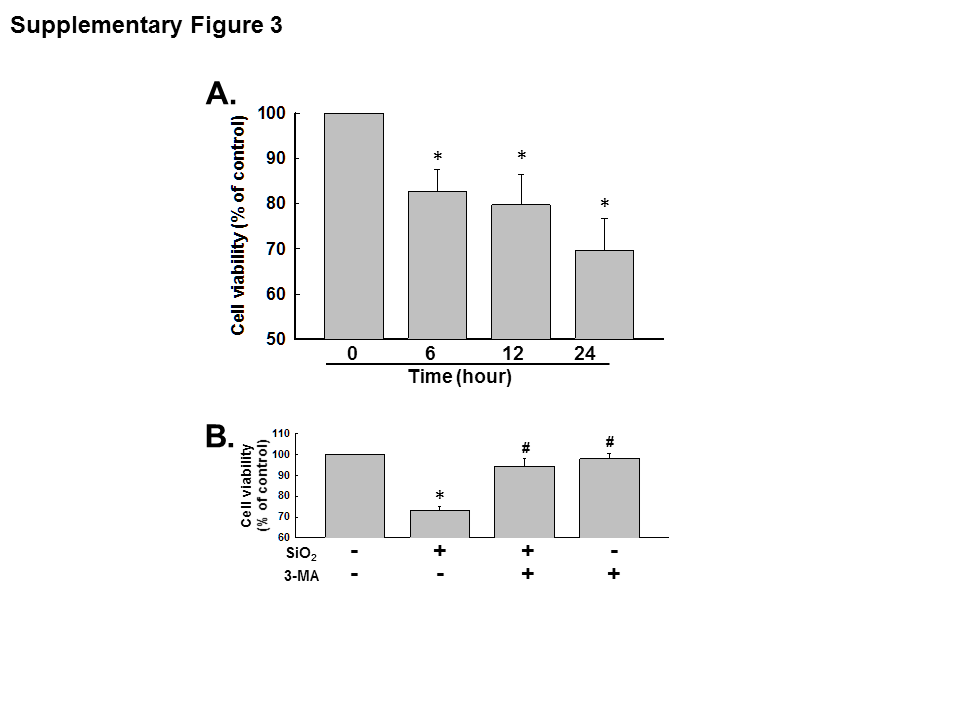

Supplement: Additional file 4: Figure S3. — Effect of SiO2 on U937 cell viability. A. MTT assay showing a time-dependent decrease in U937 cell viability induced by 50 µg/cm2 SiO2. * p < 0.05 vs the 0-hour group. B. MTT assay showing that 3-MA abolished the decrease in cell viability induced by SiO2 treatment for 24 h. * p < 0.05 vs the control group; # p < 0.05 vs the SiO2 group. [file 12989_2016_167_MOESM4_ESM.tif]

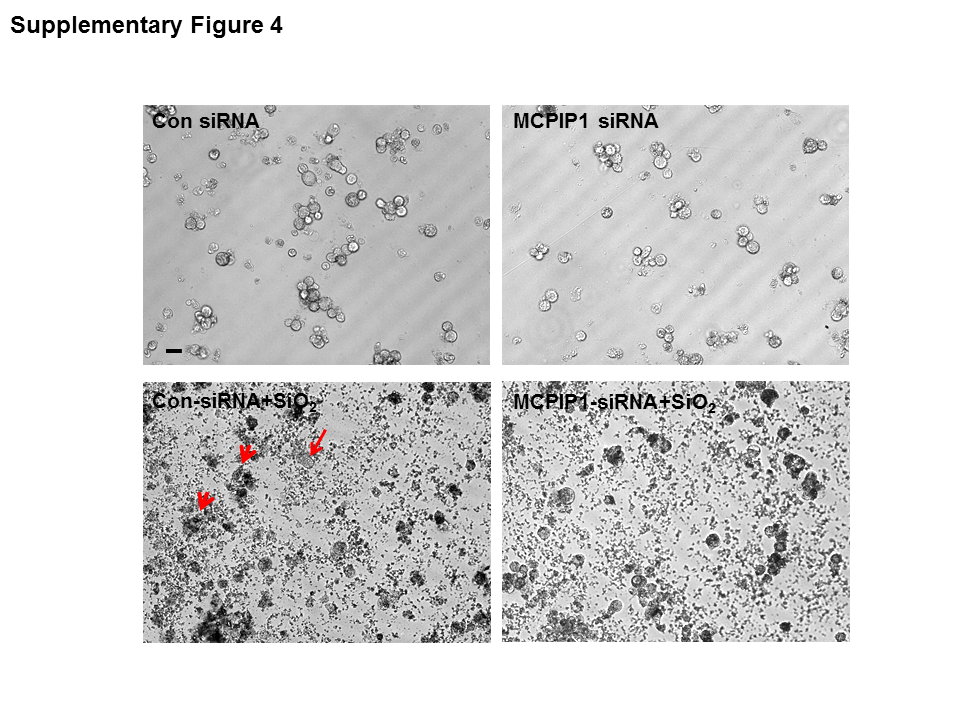

Supplement: Additional file 5: Figure S4. — Representative images showing the effects of RNAi targeting MCPIP1 on the changes in macrophage morphology induced by SiO2 treatment at 50 µg/cm2 for 24 h in U937 cells. SiO2 exposure led to morphologic changes in macrophages towards the M1 (arrow) and M2 phenotypes (arrowhead). However, knockdown of MCPIP1 significantly alleviated this effect of SiO2 treatment on macrophages (Figure S4). Scale bar = 200 µm. [file 12989_2016_167_MOESM5_ESM.tif]

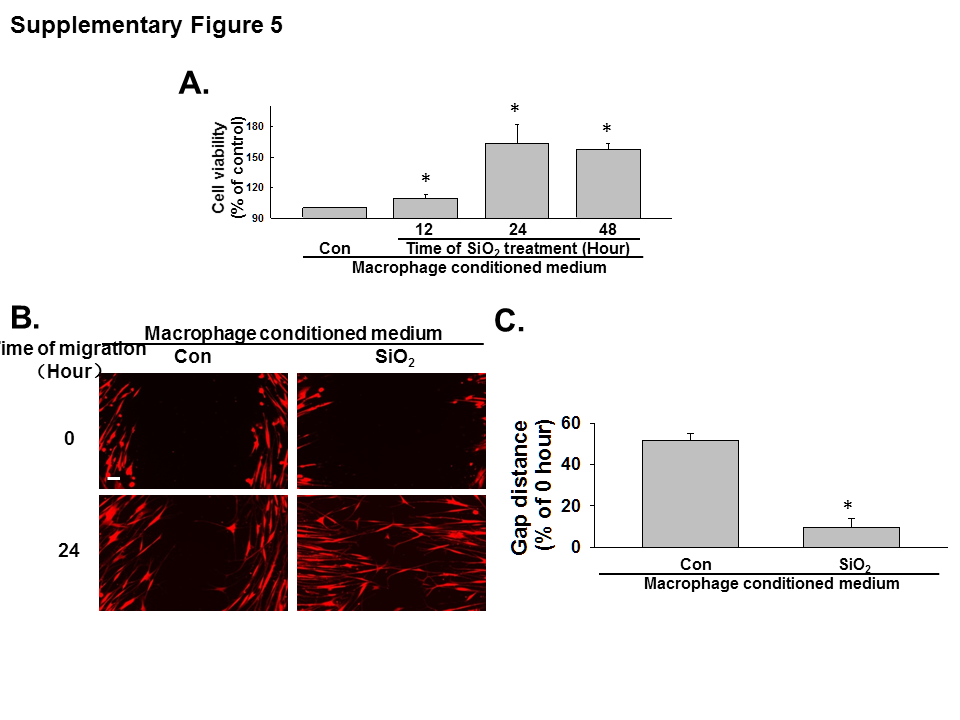

Supplement: Additional file 6: Figure S5. — Effects of conditioned medium from macrophages on HPF-a cell activation and migration. A. MTT assay showing that the conditioned medium from macrophages altered HPF-a cell viability. * p < 0.05 vs the control group. B. Representative images showing the effects of conditioned medium from macrophages exposed to SiO2 or sterile saline for 24 h on migration by RFP-labeled HPF-a cells. Scale bar = 80 µm. C. Quantification of the scratch width in six separate experiments. * p < 0.05 vs the control group. [file 12989_2016_167_MOESM6_ESM.tif]

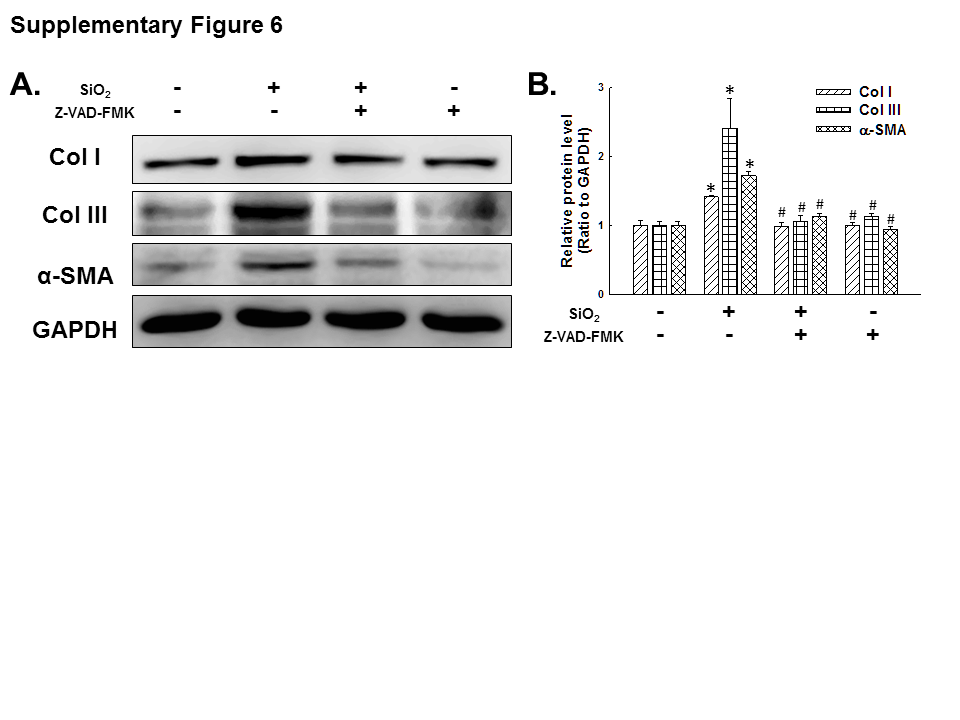

Supplement: Additional file 7: Figure S6. — Effects of macrophage apoptosis on HPF-a cell activation and migration. A. Conditioned medium was obtained from macrophages incubated in the presence or absence of Z-VAD-FMK for 1 h prior to treatment with SiO2, and this medium was applied to cultures of HPF-a cells for 24 h. Representative western blot showing the effects of conditioned medium from macrophages on the expression of collagen I, collagen III and α–SMA in HPF-a cells. B. Densitometric analyses of five separate experiments suggested that pretreatment of macrophages with Z-VAD-FMK attenuated the increases in collagen I, collagen III and α-SMA expression levels in fibroblasts induced by application of conditioned medium from macrophages that were treated with SiO2. * p < 0.05 vs the control group; # p < 0.05 vs the SiO2 group. [file 12989_2016_167_MOESM7_ESM.tif]

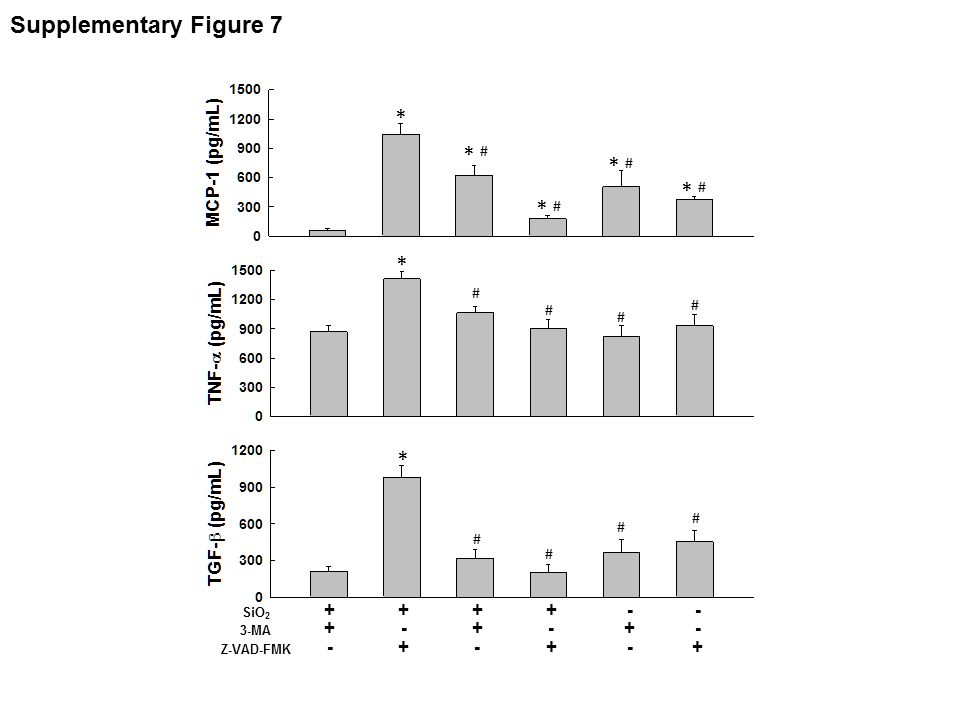

Supplement: Additional file 8: Figure S7. — Effects of SiO2 on the levels of macrophage-derived cytokines. ELISAs suggested that the upregulated expression of MCP-1, TNF-α and TGF-β in macrophages induced by SiO2 treatment for 24 h was attenuated by pretreatment with 3-MA or Z-VAD-FMK. * p < 0.05 vs the corresponding control group; # p < 0.05 vs the corresponding SiO2 group. [file 12989_2016_167_MOESM8_ESM.tif]
